# HDAC6 regulates microtubule stability and clustering of AChRs at neuromuscular junctions

**DOI:** 10.1083/jcb.201901099

**Published:** 2020-07-22

**Authors:** Alexis Osseni, Aymeric Ravel-Chapuis, Jean-Luc Thomas, Vincent Gache, Laurent Schaeffer, Bernard J. Jasmin

**Affiliations:** 1 Department of Cellular and Molecular Medicine, Faculty of Medicine, University of Ottawa, Ottawa, Ontario, Canada; 2 Éric Poulin Centre for Neuromuscular Disease, Faculty of Medicine, University of Ottawa, Ottawa, Ontario, Canada; 3 Institut NeuroMyoGene, Centre National de la Recherche Scientifique Unité Mixte de Recherche 5310, Institut National de la Santé et de la Recherche Médicale Unité 1217, Université de Lyon, Lyon, France; 4 Centre de Biotechnologie Cellulaire, Hospices Civils de Lyon, Lyon, France

## Abstract

Microtubules (MTs) are known to be post-translationally modified at the neuromuscular junction (NMJ), hence increasing their stability. To date however, the function(s) of the dynamic MT network and its relative stability in the formation and maintenance of NMJs remain poorly described. Stabilization of the MT is dependent in part on its acetylation status, and HDAC6 is capable of reversing this post-translational modification. Here, we report that HDAC6 preferentially accumulates at NMJs and that it contributes to the organization and the stability of NMJs. Indeed, pharmacological inhibition of HDAC6 protects against MT disorganization and reduces the size of acetylcholine receptor (AChR) clusters. Moreover, the endogenous HDAC6 inhibitor paxillin interacts with HDAC6 in skeletal muscle cells, colocalizes with AChR aggregates, and regulates the formation of AChR. Our findings indicate that the focal insertion of AChRs into the postsynaptic membrane is regulated by stable MTs and highlight how an MT/HDAC6/paxillin axis participates in the regulation of AChR insertion and removal to control the structure of NMJs.

## Teaser

In this article, Osseni et al. show that by controlling acetylation of microtubules at neuromuscular junctions, HDAC6 regulates the insertion and removal of acetylcholine receptors. Accordingly, these findings provide key novel insights into the mechanisms involved in the stability and structure of neuromuscular junctions.

## Introduction

The postsynaptic membrane of neuromuscular junctions (NMJs) represents a highly differentiated domain within skeletal muscle fibers (Sanes and Lichtman, 1999; Schaeffer et al., 2001; Duclert and Changeux, 1995). In addition to the accumulation of distinct myonuclei as well as structural compartmentalization of several specific cytoskeletal and membrane proteins, the postsynaptic membrane shows evidence of functional compartmentalization (Jasmin et al., 1991; Ralston, 1993; Antony et al., 1995; Ralston et al., 1999). Indeed, the postsynaptic membrane domain constitutes a sarcoplasmic region of muscle fibers specialized in the transcription, post-translational processing, and stabilization of numerous proteins of the postsynaptic membrane (Duclert and Changeux, 1995; Schaeffer et al., 2001; Schmidt et al., 2012). NMJs also contain subsynaptic networks of cortical actin filaments (F-actins), together with intermediate filaments and a specialized microtubule (MT) network (Jasmin et al., 1990, 1991; Cartaud et al., 2000; Pumplin and Strong, 1988; Dai et al., 2000; Yorifuji and Hirokawa, 1989; Sealock et al., 1989). This subsynaptic MT network (Rahkila et al., 1997; Ralston et al., 1999) contains a subpopulation of stable MTs exhibiting specific post-translational modifications associated with a higher density of total MTs (Jasmin et al., 1990; Schmidt et al., 2012). In this context, it is well known that MTs are regulated by different post-translational modifications, including acetylation and tyrosination, which are known to affect their stability. Pioneering studies performed in the early 1990s reported that MTs are in fact more acetylated in the subsynaptic domain of skeletal muscle fibers (Jasmin et al., 1990).

Acetylation of MTs is a post-translational modification of α-tubulin at lysine 40 that is regulated by a variety of acetyltransferases such as α-tubulin *N*-acetyltransferase 1 (α-TAT1), mechanosensory abnormality protein 17 (Mec-17; a homologue of α-TAT1 in *Caenorhabditis elegans*), and elongator protein 3 (Creppe et al., 2009; Akella et al., 2010; Shida et al., 2010). Conversely, the histone deacetylase (HDAC) family members sirtuin 2 and HDAC6 are responsible for deacetylation of MTs (Seigneurin-Berny et al., 2001; North et al., 2003). Over the last decade, several specific drugs have been developed against HDAC6, thereby regulating tubulin acetylation (Lee et al., 2013; Santo et al., 2012; Butler et al., 2010; Hideshima et al., 2017; Ryu et al., 2015; Pérez-Salvia et al., 2018; Zilberman et al., 2009). Interestingly, it has been shown that in many diseases such as cancers, neurological disorders, and heart and lung diseases, HDAC6 inhibition has protective effects that are associated with increased tubulin acetylation (d’Ydewalle et al., 2011; Dafinger et al., 2011; Santo et al., 2012; McLendon et al., 2014; Zhang et al., 2014a,b; Lam et al., 2013; Ota et al., 2016; Boucherat et al., 2017).

As a member of class IIb of the HDAC family, HDAC6 contains five characteristic domains including a conserved nuclear export signal at the N-terminal domain, two deacetylase catalytic domains, a cytoplasmic anchor, and an ubiquitin-binding zinc finger domain at its C-terminal domain (Hook et al., 2002; Ouyang et al., 2012). Notably, HDAC6 is the only cytoplasmic HDAC that contains a full duplication of the large class I/II HDAC-homology domain. HDAC6 is involved in at least two main biological processes, namely, deacetylation and protein ubiquitination. Indeed, in addition to deacetylation of tubulin, HDAC6 can deacetylate several other cytoplasmic substrates such as cortactin and the heat shock protein 90 (HSP90; Kovacs et al., 2005; Zhang et al., 2007). Moreover, HDAC6 interacts with components of the ubiquitin proteasome pathway through its ubiquitin-binding domain and can thus play a critical role in the cellular response to misfolded and aggregated proteins (Boyault et al., 2007a,b; Lee et al., 2010; Hubbert et al., 2002; Gao et al., 2010; Kawaguchi et al., 2003; Valenzuela-Fernández et al., 2008; Zhang et al., 2003).

The postsynaptic compartment of muscle fibers is indeed specialized transcriptionally. Within this region of muscle fibers, a specific subset of genes is induced by the presence of the nerve, affecting selectively their transcription in myonuclei underlying the synapse. At NMJs, the acetylcholine receptor (AChR) accumulates at a density of up to ∼15,000 molecules per square micrometer (Laufer and Changeux, 1989; Salpeter and Loring, 1985), whereas only a few AChRs are found per square millimeter in extra-synaptic regions (Fertuck and Salpeter, 1976). Once AChR mRNAs and proteins are synthesized subsynaptically, their precise targeting and efficient transport to the synaptic membrane become crucial for maintaining optimal functional communication between the nerve and muscle fiber. In sharp contrast to our knowledge of the transcriptional regulatory events that control expression of synaptic genes, there is relatively little information concerning the events that lead to insertion of newly synthesized AChRs within the postsynaptic membrane. One possible mechanism involves focal transport via the MT network oriented toward the subsynaptic membrane. In muscle fibers, this local insertion likely requires MTs interconnected with Golgi elements (Jasmin et al., 1995; Ralston et al., 1999).

In neurons, the MT network is known to participate in neuronal polarization, axonal transport, axonal growth, and regeneration (Murillo and Mendes Sousa, 2018; Janke and Kneussel, 2010). Accordingly, inhibition of HDAC6 in cortical and dorsal root ganglion neurons enhances tubulin acetylation and promotes axon growth, thereby demonstrating the need for stable MTs within axons for cargo delivery (Rivieccio et al., 2009). By contrast, the functional roles of the MT network within the postsynaptic domain of muscle fibers as well as the implications of its acetylation status are still poorly understood. In the present work, we thus set out to examine these central questions by (a) determining the mechanisms that control acetylation of MTs within the postsynaptic sarcoplasm and (b) elucidating the impact of HDAC6 in AChR clustering and NMJ organization.

## Results

### MTs and acetylated tubulin are enriched at NMJs

**Figure 1.**
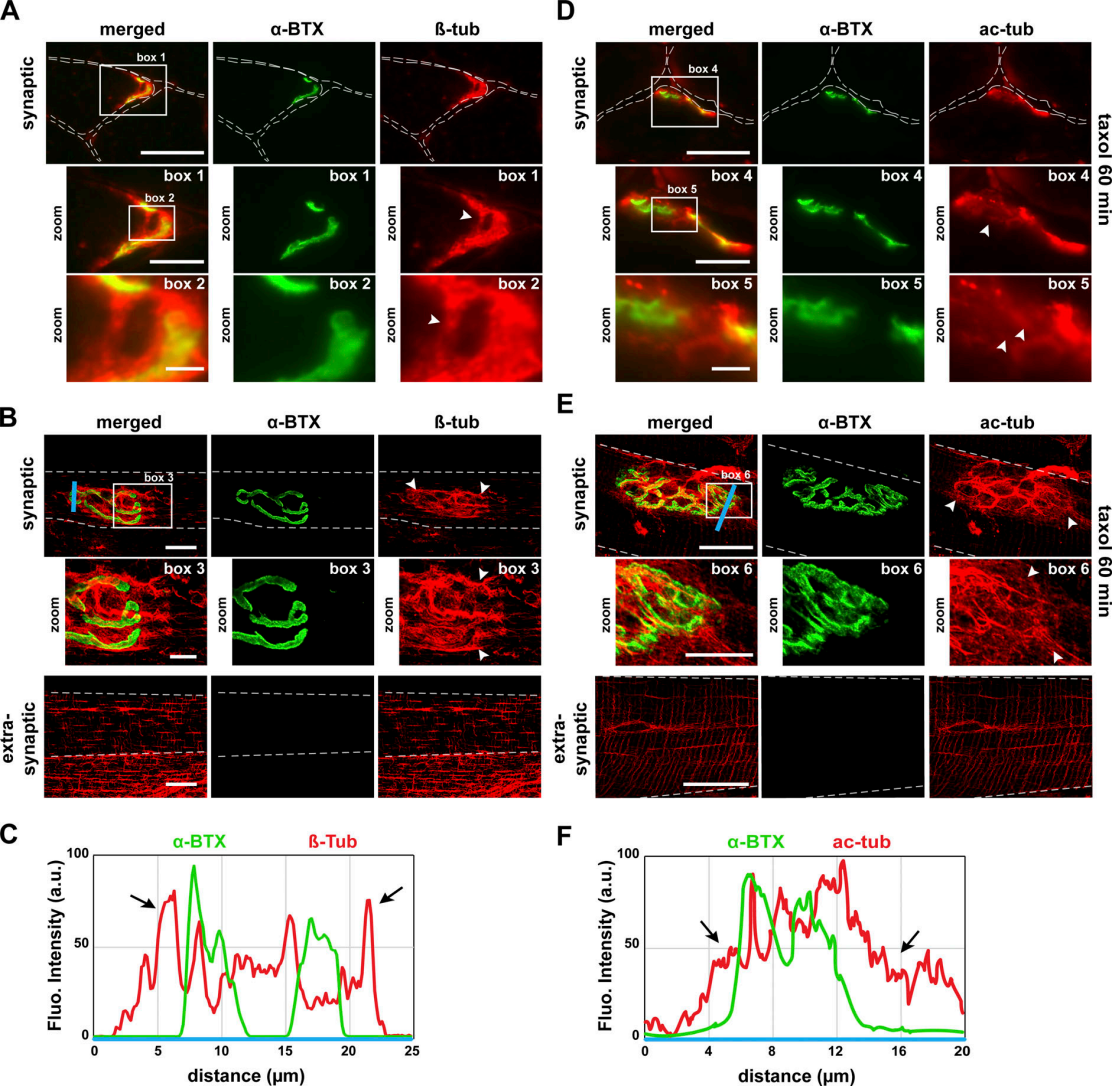
**MT network and acetylated tubulin localize at the NMJ.** **(A, B, D, and E)** Cross sections (A, D) and isolated fibers (B, E) of TA muscle from 2-mo-old WT mice in the presence (D, E) or absence (A, B) of taxol were double-stained with an antibody against α-tubulin to label MT network (A and B; β-tub, in red) or against acetylated tubulin to label stable MT (D and E; ac-tub, in red) and with α-BTX–A488 (in green) to label NMJs. **(C and F)** The fluorescence intensity of each staining was plotted as a function of the distance (based on the blue line scans in B and E, respectively). Arrowheads and arrows show enrichment of the MT network surrounding NMJs. Dashed lines indicate edges of cells. **(A, B, D, and E)** Scale bars: 25 µm; inset magnifications (boxes 1, 3, 4, and 6): 10 µm; (boxes 2 and 5): 2 µm. Fluo., fluorescence; β-tub, β-tubulin; ac-tub, acetylated tubulin.

The postsynaptic domain of skeletal muscle fibers is known to be associated with a dense MT network (Ralston et al., 1999; Jasmin et al., 1991; Camus et al., 1998; Connolly, 1985; Rahkila et al., 1997). Therefore, we first analyzed by fluorescence microscopy the spatial organization of the MT network in both dissociated fibers and cross sections of tibialis anterior (TA) muscles. NMJs were labeled with α-bungarotoxin–Alexa-488 (α-BTX–A488), and MTs were visualized through β-tubulin immunostaining. In agreement with previous data obtained on mouse soleus (Ralston et al., 1999) and flexor digitorum brevis muscles (Rahkila et al., 1997) as well as with chick anterior latissimus dorsi muscle (Jasmin et al., 1990), we observed a strong tubulin labeling at NMJs (Fig. 1 A, box 1 and 2; and Fig. 1 B, box 3). More specifically, our results show that NMJs are surrounded by a dense network of tubulin (see line scan in Fig. 1 C). In addition, the organization of the postsynaptic MT network areas is clearly distinct from that seen in extra-synaptic compartments where MT networks have a specific grid-like organization with longitudinal, transverse, and perinuclear MTs (Fig. 1 B, extra-synaptic panels).

It was previously demonstrated that post-translationally modified tubulin (tyrosination and acetylation) is enriched in subpopulations of stable, subsynaptic MTs (Schmidt et al., 2012; Jasmin et al., 1990). To confirm that acetylated tubulin is indeed preferentially located within the subsynaptic MT network of mammalian muscle fibers, we performed fluorescence experiments on TA muscle cross sections in the presence of taxol, which is a powerful stabilizing agent of the MT network. In agreement with previous findings from Jasmin et al. (1990) using taxol-treated chick muscle, we observed marked acetylated-tubulin staining within postsynaptic regions, indicating that MTs are stabilized at the NMJ (Fig. 1 D, box 4 and 5; and Fig. 1 E, box 6). As described for the MT network labeled with a β-tubulin antibody, acetylated tubulin also surrounds the NMJs (see line scan in Fig. 1 F). Together, these data indicate that MTs are enriched and acetylated within postsynaptic domains of mouse muscle fibers.

### HDAC6 localizes at the NMJ in vivo and with AChR clusters in vitro

Acetylation of MTs is a post-translational modification of α-tubulin at lysine 40. Acetylation of tubulin is in a constant dynamic balance between acetylation and deacetylation. Among all tubulin acetyltransferases expressed in mammalian cells, α-TAT1 is known to be the major one in mice (Kalebic et al., 2013), whereas HDAC6 is the main tubulin deacetylase (Hubbert et al., 2002; Seigneurin-Berny et al., 2001). We thus hypothesized that either α-TAT1 or HDAC6 has a role in controlling tubulin acetylation within postsynaptic membrane domains. To this end, we examined their localization at NMJs by immunofluorescence.

**Figure 2.**
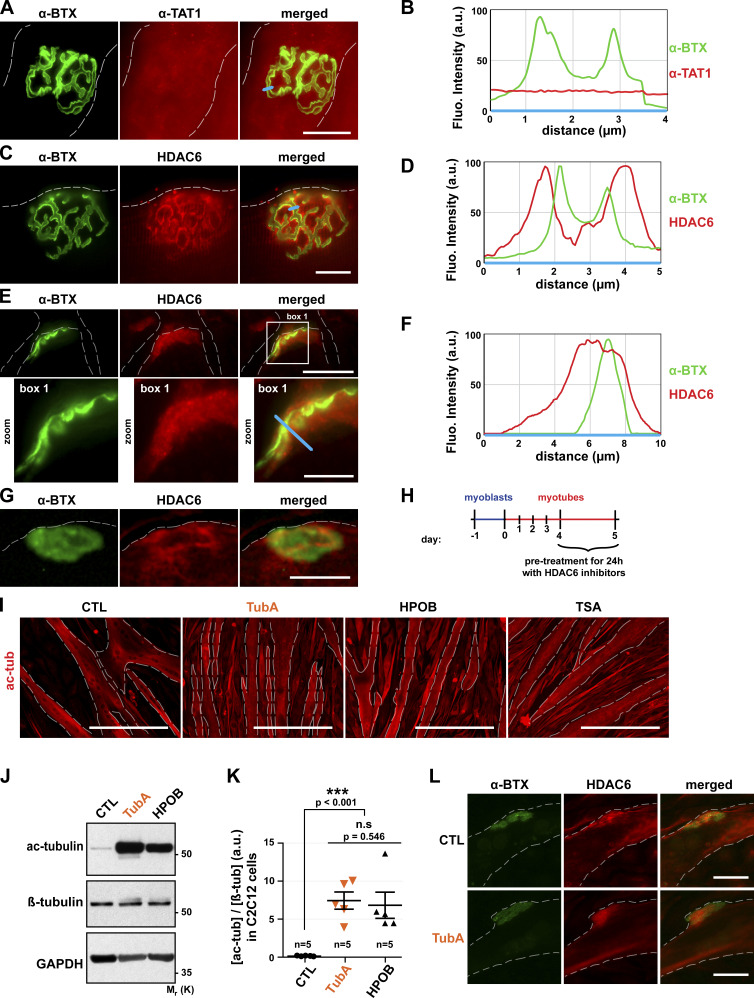
**In muscle cells, HDAC6 is enriched within postsynaptic domains, and its inhibition increases tubulin acetylation.** **(A, C, E, G, I, and L)** Isolated fibers (A and C) and cross sections (E) of TA muscles from 2-mo-old mice and 5-d-old C2C12 myotubes (G and L) were stained in red with an antibody against α−TAT1 (A), HDAC6 (C, E, G, and L), or acetylated tubulin (ac-tub; I). NMJs and AChR clusters were labeled with α-BTX–A488 (A, C, E, G, and L; in green). **(B, D, and F)** The fluorescence intensity of each staining was plotted as a function of the distance (based on the blue line scans in A, C, and E, respectively). **(H)** Schematic representation of the experimental time course. **(I–L)** 4-d-old myotubes pretreated for 24 h with different HDAC6 inhibitors TubA (5 µM) and HPOB (5 µM), with the pan-HDAC inhibitor TSA (0.1 µM), or with DMSO (CTL, 1 µl). **(J)** Representative Western blots showing acetylated tubulin (Ac-tubulin) and β-tubulin expressions. GAPDH was used as a loading control. **(K)** Quantification of acetylated tubulin protein levels normalized with β-tubulin (*n* = number of independent Western blots quantified; 5). Graphs show means ± SEM. ***, P < 0.001; n.s not significant; Mann-Whitney *U* test. Dashed lines indicate edges of cells. Scale bars: 25 µm (A, C, E, G, and L); 400 µm (I); inset magnifications (E; box 1): 10 µm. Fluo., fluorescence; β-tub, β-tubulin, n.s, not significant; M_r_(K), relative molecular weight in kiloDalton; ac-tub and ac-tubulin, acetylated tubulin.

First, we performed immunofluorescence studies in dissociated TA muscle fibers using an antibody specific for α-TAT1. In TA dissociated fibers, NMJs labeled with α-BTX–A488 showed the characteristic pretzel-like morphology (Fig. 2, A and C). As revealed by these experiments, α-TAT1 is not significantly enriched at NMJs (Fig. 2 A) as observed on the line scan (Fig. 2 B). By contrast, similar immunofluorescence experiments clearly showed a postsynaptic enrichment of HDAC6 (Fig. 2 C), with HDAC6 staining encompassing AChR labeling (Fig. 2 D). We confirmed these observations in cross sections of TA muscles (Fig. 2 E). In fact, HDAC6 accumulates in the postsynaptic region, surrounding the primary gutters, and partially overlaps with AChR as shown by the yellow areas on merged images. Collectively, these observations suggest that the acetylation status of tubulin within the postsynaptic compartments of muscle fibers is preferentially regulated by HDAC6.

To further examine the localization of HDAC6 with respect to AChRs, we also assessed its localization in differentiated C2C12 myotubes in culture (Fig. 2 G). Previous work reported that AChR clusters can form spontaneously when myotubes are cultured on plates coated with laminin (Kummer et al., 2004). These AChR aggregates display several characteristic features of the mature postsynaptic apparatus, including colocalization of multiple postsynaptic proteins and clustering of subjacent myonuclei (Bruneau et al., 2005a, 2005b). Accordingly, this culture system represents a relevant model to study postsynaptic membrane organization. We therefore differentiated C2C12 myotubes on Matrigel-coated plates, a matrix that contains laminin. Immunofluorescence experiments were performed on 5-d–differentiated myotubes. As expected, structured AChR clusters formed at the surface of these myotubes (Fig. 2, G and L). In addition and in agreement with our in vivo data obtained with TA muscle fibers (see above), we also observed in these experiments an accumulation of HDAC6 at AChR clusters (Fig. 2 G). Taken together, these results show that in dissociated muscle fibers as well as in myotubes in culture, HDAC6 accumulates in the vicinity of AChR clusters.

### HDAC6 inhibition increases tubulin acetylation in muscle cells

**Figure S1.**
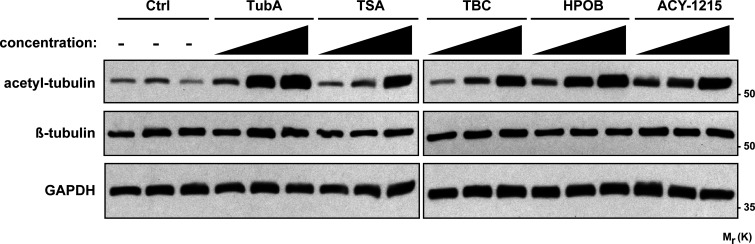
**Expression of tubulin acetylated via HDAC6 inhibitors in HEK293 cells.** HEK293 cells were treated for 24 h with different HDAC6 inhibitors at different concentrations: TubA (0.05, 0.5, and 5 µM), HPOB (0.05, 0.5, and 5 µM), ACY-1215 (0.01, 0.1, and 0.5 µM), and TBC (0.05, 0.5, and 5 µM) with a pan-HDAC inhibitor: TSA (0.001, 0.01, and 0.1 µM); or with DMSO (Ctrl, control; 0.1, 1, and 10 µl). Representative Western blots showing acetylated tubulin (acetyl-tubulin) and β-tubulin expressions. GAPDH was used as a loading control. Mr(K), relative molecular weight in kiloDalton.

To examine the role of HDAC6 in regulating the MT network at NMJs, we used tubastatin A (TubA) and N-hydroxy-4-(2-[(2-hydroxyethyl)(phenyl)amino]-2-oxoethyl)benzamide (HPOB), two specific inhibitors of HDAC6 activity. In these experiments, the activity of HDAC6 was evaluated by measuring the levels of acetylated tubulin. 4-d-old myotubes were treated with each one of these drugs (Fig. 2 H). 24 h later, the level of tubulin acetylation was assessed by immunofluorescence microscopy and Western blotting. As shown in Fig. 2, I and J, TubA and HPOB both caused a large increase in α-tubulin acetylation in C2C12 myotubes. Quantification of the relative level of acetylated tubulin by Western blot showed an ∼7% increase following treatments with these specific HDAC6 inhibitors (P value <0.001 compared with control [CTL]; Fig. 2 K). Furthermore, suppression of HDAC6 activity by the specific inhibitors TubA, tubacin (TBC), HPOB, and rocilinostat (ACY-1215) resulted in an increase in MT acetylation (see Fig. S1), as previously observed (Haggarty et al., 2003; Lee et al., 2013; Butler et al., 2010; Hubbert et al., 2002). Together, these data indicate that HDAC6 is a key α-tubulin deacetylase in muscle cells, and those specific drugs are able to efficiently inhibit its deacetylase activity, thereby impacting the levels of acetylated tubulin in muscle cells.

In additional experiments, we also investigated whether the inhibition of HDAC6 had an effect on the localization of HDAC6. For this, we treated 4-d-old myotubes cultured on Matrigel-coated plates with TubA for 24 h. As shown in Fig. 2 L, TubA did not affect the colocalization of HDAC6 with AChR clusters. These results show that increased MT acetylation via the inhibition of HDAC6 does not affect the localization of HDAC6 at AChR clusters.

### Increased tubulin acetylation via HDAC6 inhibition protects against MT disorganization

**Figure 3.**
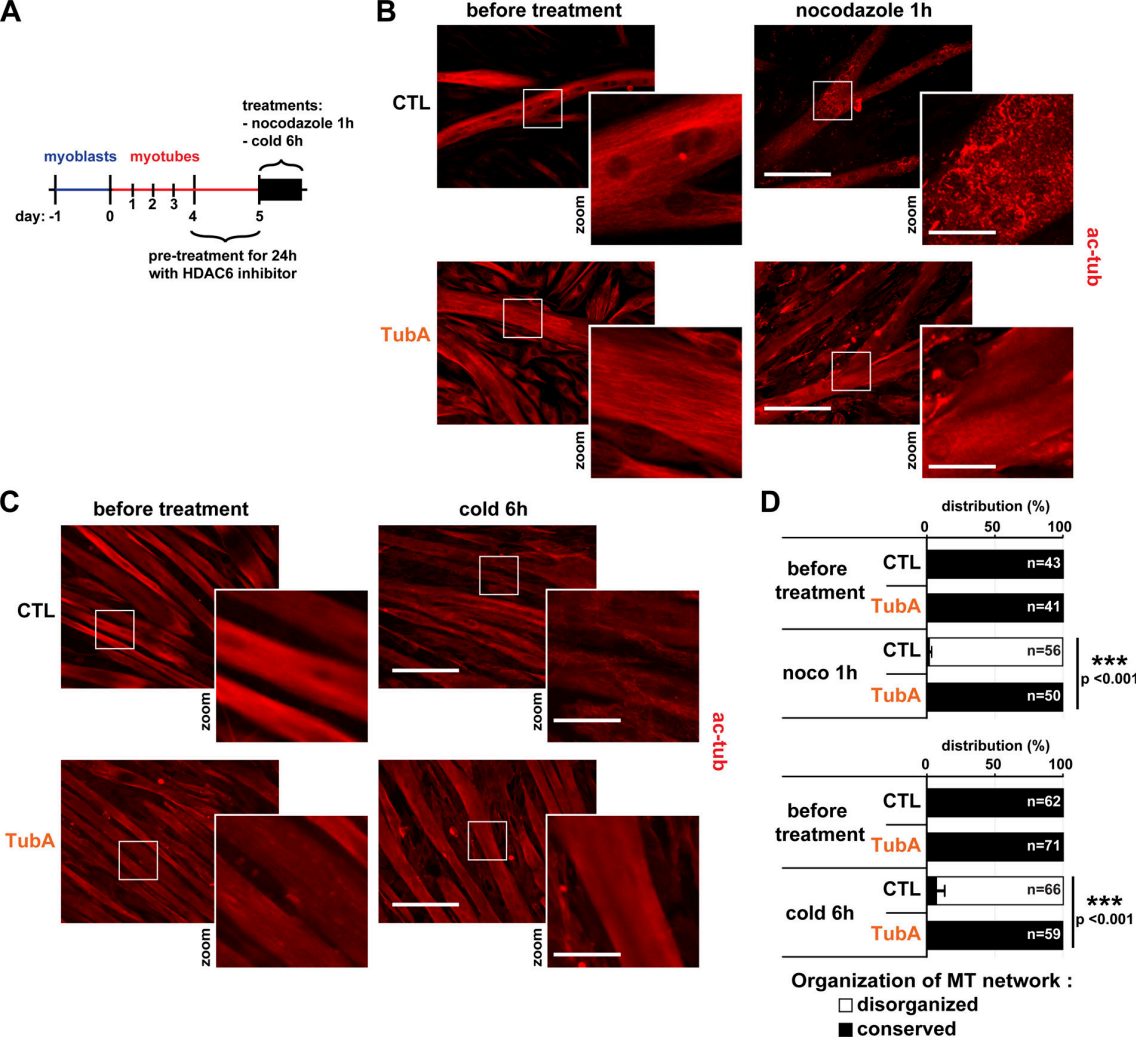
**Specific inhibition of HDAC6 protects against MT disorganization.** **(A)** Schematic representation of the experimental time course. **(B and C)** 4-d-old C2C12 myotubes were pretreated with the specific HDAC6 inhibitor TubA (5 µM) or with DMSO (CTL; 1 µl). After 24 h of pretreatment, cells were either treated for 1 h with nocodazole (10 µM) or put on ice for 6 h (cold treatment). C2C12 cells were stained with an antibody against acetylated tubulin (ac-tubulin, in red). **(D)** Quantification of the distribution of the organization of the MT network in myotubes (three independent experiments for each condition; *n* = number of myotubes counted; between 41 and 71 myotubes). Means ± SEM. ***, P < 0.001; Mann-Whitney *U* test. **(B and C)** Scale bars: 200 µm; inset magnifications (zoom): 50 µm. ac-tub, acetylated tubulin.

As shown above, inhibition of HDAC6 activity enhanced MT acetylation in myotubes (Fig. 2). We thus wondered whether the increase in tubulin acetylation induced by pharmacological inhibition of HDAC6 could impact the stability of the MT network in muscle cells. To evaluate this (Fig. 3 A), 4 d–differentiated myotubes were treated with TubA for 24 h to achieve a high level of tubulin acetylation (Fig. 3, B and C, lower panels). Then, the HDAC6 inhibitor was removed, and myotubes were exposed to either nocodazole for 1 h (Fig. 3 B) or to a 6-h cold treatment at 4°C (Fig. 3 C). Both of these treatments are known to disorganize the MT network by depolymerizing individual MTs (Tassin et al., 1985; Lieuvin et al., 1994). To visualize these effects, myotubes were labeled with an anti–acetylated tubulin antibody, and the MT organization was analyzed by immunofluorescence. For quantifications, MT organization in myotubes was categorized into two distinct states, either (a) “conserved” with MT treads, in which the MT network is well organized, or (b) “disorganized,” in which MTs are depolymerized. As expected, both cold and nocodazole efficiently depolymerized MTs (Fig. 3, B and C, upper right panels). In cold- and nocodazole-treated myotubes, the MT network was almost completely disorganized (∼95% of control; P value <0.001 compared with CTL; Fig. 3 D). Pretreatment of myotubes with TubA prevented the disorganization of MTs in both cold- and nocodazole-treated myotubes, thereby indicating that networks of acetylated MTs can resist depolymerization-inducing treatments. Therefore, these data show that specific inhibition of HDAC6 protects against MT disorganization.

### Inhibition of HDAC6 reduces the size of AChR clusters

**Figure 4.**
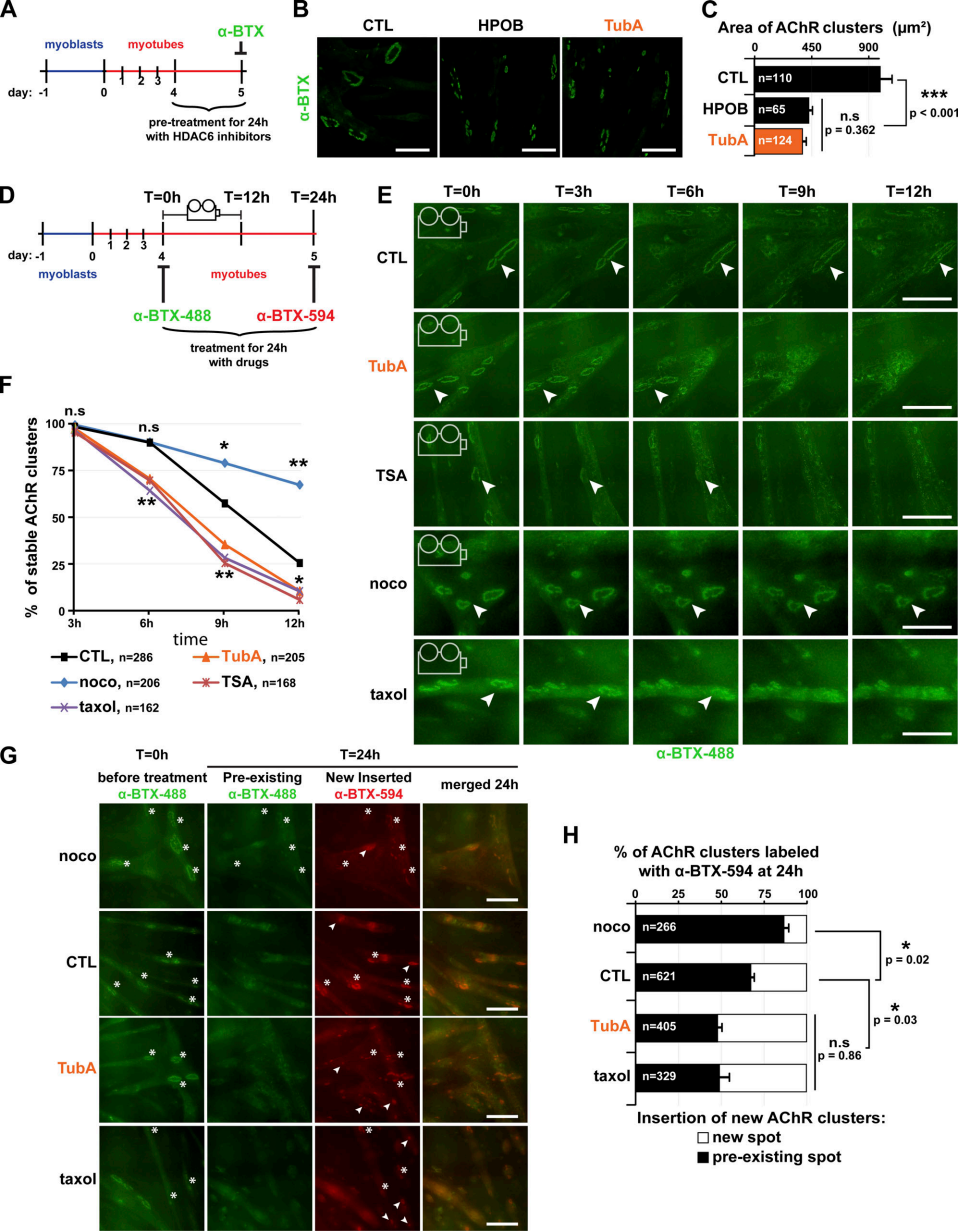
**Inhibition of HDAC6 regulates the clustering of AChR complexes by organizing the MT network.** **(A)** Schematic representation of the experimental time course for B and C. **(B)** 4-d-old C2C12 myotubes were pretreated for 24 h with HDAC6 inhibitors HPOB (5 µM) and TubA (5 µM) or with DMSO (CTL; 1 µl). AChR clusters were labeled with α-BTX–A488 (in green). **(C)** Quantifications of AChR cluster areas of three independent experiments (*n* = number of AChR clusters counted, between 65 and 124). **(D)** Schematic representation of the experimental time lapse imaging for E–H. **(E and F)** AChR clusters of 4-d-old C2C12 myotubes were labeled with α-BTX–A488 (in green). Myotubes were treated with either DMSO (CTL; 1 µl, curve in black), TubA (5 µM, curve in orange), TSA (0.1 µM, curve in red), nocodazole (10 µM, curve in blue), or taxol (10 µM, curve in purple) and imaged over 12 h (Video 1 and Video 2). Representative images are shown every 3 h; arrowheads point to representative AChR clusters. **(F)** Quantification of three independent experiments showing the disappearance of AChR clusters at the surface of myotubes between 3 and 12 h (*n* = total number of AChR clusters counted, between 162 and 286). *, P < 0.05; **, P < 0.01; n.s, not significant; two-way ANOVA. **(G)** 4-d-old myotubes were labeled with α-BTX–A488 (in green), then treated with drugs for 24 h, and finally labeled with α-BTX–A594 (in red) at day 5. Insertions of new AChR clusters are shown in preexisting AChR clusters (asterisks) or in new localizations (arrowheads). **(H)** Quantification showing the distribution of new AChR clusters at day 5 (three independent experiments; *n* = total number of AChR clusters counted, between 266 and 621). **(C and H)** Means ± SEM. *, P < 0.05; ***, P < 0.001; Mann-Whitney *U* test. **(B, E, and G)** Bars: 100 µm. noco, nocodazole; n.s, not significant; T, time.

**Figure S2.**
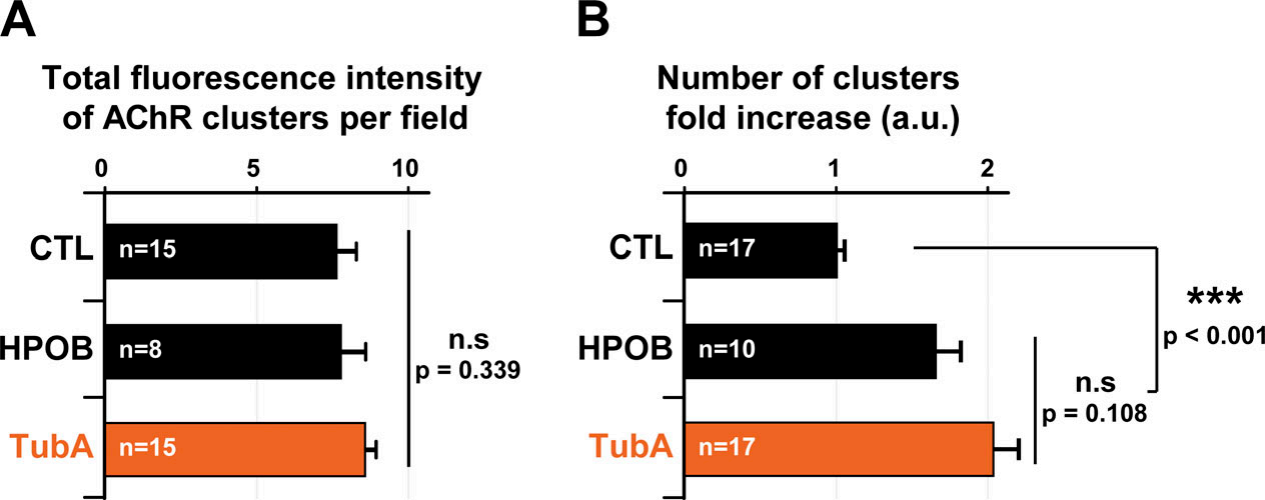
**Quantifications of number of AChR clusters in C2C12 cells.** 4-d-old C2C12 myotubes were pretreated for 24 h with HDAC6 inhibitors HPOB (5 µM) and TubA (5 µM) or with DMSO (CTL; 1 µl). **(A)** Quantification of the total fluorescence intensity of AChR clusters per field; *n* = number of fields of 0.15 mm^2^ counted, between 8 and 15. **(B)** Quantification of the number of clusters per field; *n* = number of fields of 0.15 mm^2^ counted, between 10 and 17. Each quantification result from three independent experiments. Means ± SEM. ***, P < 0.001; n.s, not significant; Mann-Whitney *U* test.

Based on the above findings, we hypothesized that pharmacological inhibition of HDAC6 would impact postsynaptic domains. Therefore, we examined the effect of HDAC6 inhibition on AChR clusters. In a first set of experiments, we asked whether MT stability is essential for the maintenance of AChR clusters. 4 d–differentiated myotubes were treated with each one of the specific inhibitors of HDAC6 for 24 h (Fig. 4 A). At the end of the drug treatment, AChR clusters were labeled with α-BTX–A488 (Fig. 4 B), and their area was measured. AChR clusters were markedly smaller (∼50%; P value <0.001) following HDAC6 inhibition (with TubA or HPOB) compared with untreated myotubes (Fig. 4 C). Moreover, while total fluorescence of AChR clusters remained unchanged (Fig. S2 A), the number of AChR clusters was increased by ∼1.7-fold to twofold in HPOB- and TubA-treated myotubes, respectively (P value <0.001 compared with CTL; Fig. S2 B). These data indicate that HDAC6 inhibition promotes either the fragmentation of existing AChR clusters or the disappearance of existing clusters with insertion of novel ones. In either scenario, the results suggest a clear functional link between MT acetylation and maintenance of AChR clusters.

### HDAC6 inhibition increases both the disappearance rate of AChR clusters and the formation of new clusters via MTs

The observed decrease in the size of AChR clusters can occur through either a reduction of AChR insertion into the membrane or an acceleration of AChR removal from the clusters. To distinguish between these two possibilities, the series of above experiments was repeated, but this time, AChR clusters were first stained with α-BTX–A488 in green at the beginning of the incubation period (Fig. 4 D). AChR clusters were then imaged every 30 min for 12 h (see Video 1). We observed that the disappearance of AChR clusters over time was accelerated in the presence of HDAC6 inhibitors (Fig. 4, E and F). As expected in control conditions (Bruneau et al., 2005b), ∼50% of AChR clusters vanished within ∼10 h (P value <0.001). In TubA-treated myotubes, AChR clusters disappeared faster (∼8 h; Fig. 4 F). After 6 h of treatment, ∼30% of α-BTX–A488–labeled AChR clusters had vanished in both trichostatin A- (TSA; a pan HDAC inhibitor) and TubA-treated cells (P value = 0.007 compared with CTL), whereas only ∼10% were lost in control myotubes (P value <0.001; Fig. 4 F).

**Video 1.** **Dynamics of AChR clusters with HDAC6 inhibitors in C2C12 cells.** 4-d-old myotubes were treated either with TubA (5 µM), TSA (0.1 µM), or a vehicle (DMSO, 1 µl). AChR clusters were labeled with α-BTX–A488 (in green) as described in Fig. 4 E. Using cell live imaging acquisition, AChR clusters were imaged every 30 min for 12 h at 37°C on an IncuCyte ZOOM system. In six-well plates, 16–25 images were taken by well per condition. Imaging was performed every 30 min for 12 h. The video represents 24 images in 5 sec.

We further explored the role of the MT network in the maintenance of AChR clusters using other drugs that also modify MT stability. For these, AChR clusters were first labeled as above, but then myotubes were treated with classic drugs that affect the stability of the MT network, namely, nocodazole (a destabilizing agent) and taxol (a stabilizing agent, similarly causing hyperacetylation of tubulin following HDAC6 inhibition). In taxol-treated conditions, disappearance of AChR clusters was similar to that seen in TubA- or TSA-treated myotubes (Fig. 4 E). After 12 h of treatment, for example (see Video 2), ∼90% of AChR clusters disappeared in taxol-treated myotubes, similar to what was observed in TubA conditions (P value = 0.011 compared with CTL; Fig. 4 F). In contrast, AChR clusters were more stable under depolymerizing conditions using nocodazole (Fig. 4 E). Indeed, after 12 h of treatment, only ∼30% of AChR clusters had disappeared in nocodazole-treated myotubes (P value <0.001 compared with CTL) versus ∼75% in control conditions (Fig. 4 F).

**Video 2.** **Dynamics of AChR clusters with MT drugs in C2C12 cells.** 4-d-old myotubes were treated with either nocodazole (10 µM) or taxol (10 µM). AChR clusters were labeled with α-BTX–A488 (in green) as described in Fig. 4 E. Using cell live imaging acquisition, AChR clusters were imaged every 30 min for 12 h at 37°C on an IncuCyte ZOOM system. In six-well plates, 16–25 images were taken by well per condition. Imaging was performed every 30 min for 12 h. The video represents 24 images in 5 sec.

To visualize the localization of newly inserted AChR molecules, AChR clusters were stained and imaged again at the end of the drug treatments using red α-BTX–A594 (Fig. 4, D and G). After 24 h, we observed that the TubA treatment allowed for insertion of new AChR molecules (labeled in red) in regions that did not colocalize with preexisting clusters (labeled in green). Approximately 50% of AChR clusters in TubA-treated myotubes were localized in new areas (P value = 0.03 compared with CTL; Fig. 4 H). This level of insertion into new regions of the sarcolemma was similar to that observed in taxol-treated myotubes. By contrast, only ∼30% of AChR clusters localized to new areas in control conditions (P value <0.001; Fig. 4 H). Finally, nocodazole induced effects opposite to those of TubA and taxol on the appearance of AChR clusters, with only ∼10% of AChR clusters being present in new spots after 24 h (P value = 0.02 compared with CTL). These data highlight that MT acetylation via HDAC6 inhibition participates in the spatial organization of AChR insertion into new clusters. More specifically, the increase in MT acetylation via HDAC6 inhibition accelerates removal of AChR from preexisting clusters and the insertion of novel AChR clusters into distinct locations along myotubes.

### Paxillin colocalizes with AChR at NMJs and regulates MT acetylation

**Figure 5.**
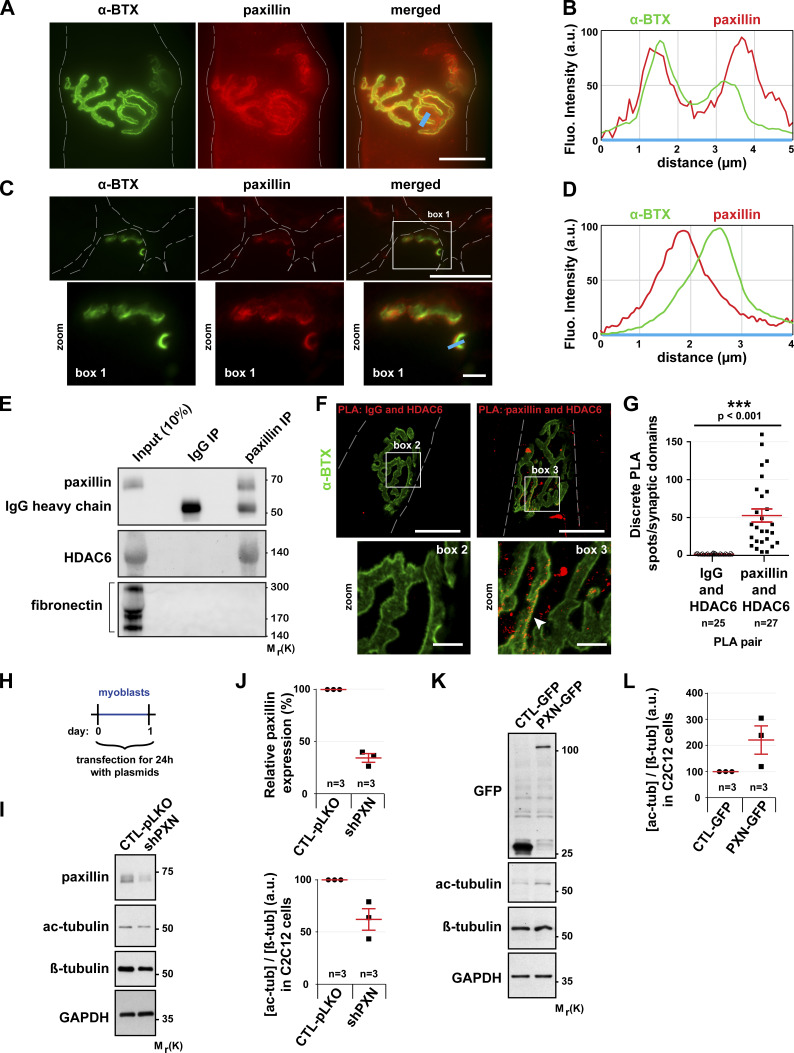
**In muscle cells, paxillin is present at the NMJ and promotes the regulation of MT acetylation.** **(A and C)** Isolated fibers (A) and cross sections (C) of TA muscles from 2-mo-old mice were double-stained with an antibody against paxillin (in red) and with α-BTX–A488 (in green). **(B and D)** The fluorescence intensity of each staining was plotted as a function of the distance (based on the blue line scans in A and C, respectively). The green curve corresponds to α−BTX–A488 staining and the red curve to paxillin staining. **(E)** Western blot showing the co-immunoprecipitation of endogenous HDAC6 and paxillin in TA muscle cells. **(F and G)** Representative images (F) and quantitation (G) of a PLA performed in isolated fibers of TA muscle with protein-specific antibody pairs as indicated. Cells were counterstained with α-BTX–A488 in green (*n* = number of synaptic domains quantified). PLA-positive spots are shown in red. The arrowhead shows the colocalization of α-BTX and PLA. Means ± SEM. ***, P < 0.001; Mann-Whitney *U* test. **(H)** Schematic representation of the experimental time course. **(I–L)** Myoblasts were transfected with either shRNA-Control (pLKO), shRNA against paxillin (shPXN), GFP alone, or PXN-GFP for 24 h. **(I and K)** Representative Western blots showing endogenous paxillin, acetylated tubulin (ac-tubulin), GFP, and β-tubulin expression. GAPDH was used as a loading control. **(J and L)** Quantification of acetylated tubulin protein level, normalized to β-tubulin. Paxillin protein level quantification was standardized to GAPDH. Means ± SEM (*n* = number of Western blots quantified). The baseline was established to 1 for the control condition for each individual Western blot. Dashed lines indicate edges of cells. Scale bars: 25 µm (A, C, and F); inset magnifications (C and F; boxes 1, 2, and 3) 5 µm. Fluo, fluorescence; β-tub, β-tubulin; IP, immunoprecipitation; Mr(K), relative molecular weight in kiloDalton; ac-tub and ac-tubulin, acetylated tubulin.

Paxillin is a key component of the cellular adhesome (Zaidel-Bar et al., 2007). It regulates the growth and the stability of focal adhesions (Webb et al., 2004; Deakin and Turner, 2011). Moreover, paxillin was reported to be enriched at NMJs (Turner et al., 1991). Labeling of dissociated fibers of TA muscles with an anti-paxillin antibody and α-BTX–A488 confirmed that paxillin indeed accumulates at NMJs (Fig. 5, A and C), where it precisely colocalizes with α-BTX–A488 labeling (see line scans in Fig. 5 B). Moreover, paxillin and AChR staining of muscle cross sections showed that paxillin localizes just below AChR in the postsynaptic domain (see line scan in Fig. 5 D).

**Figure S3.**
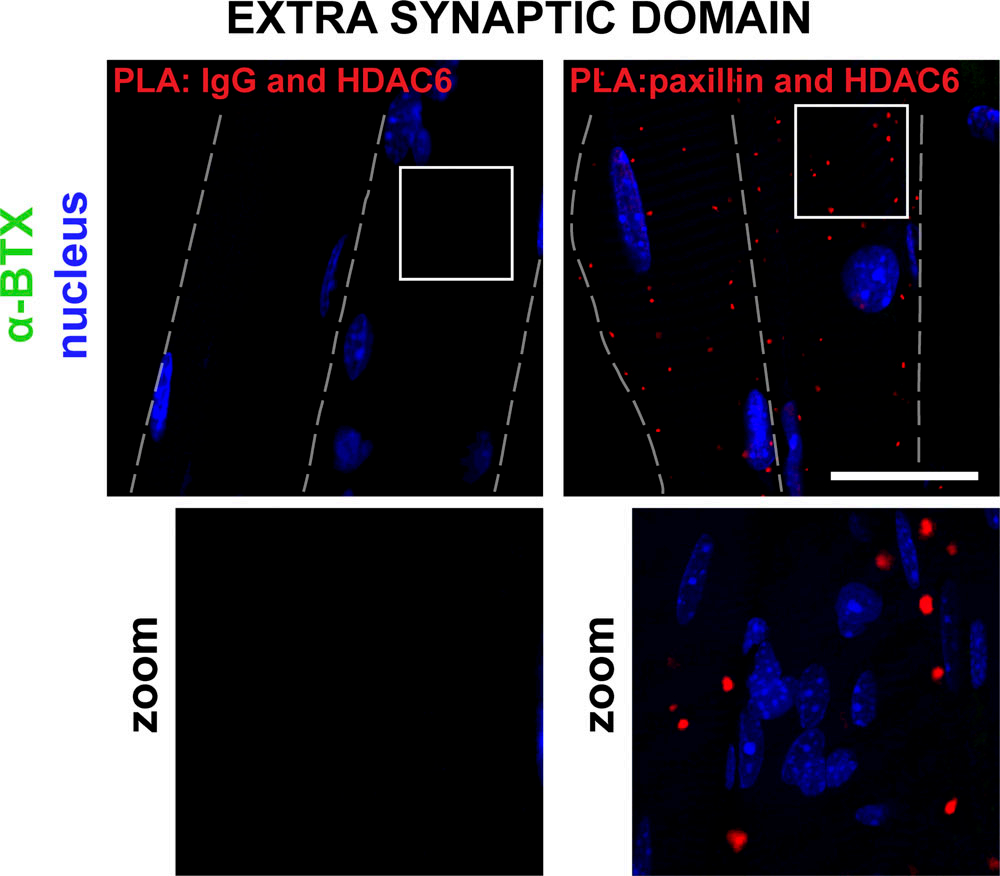
**PLA at the extra-synaptic domain in TA muscle.** Representative images of a PLA performed in isolated fibers of TA muscle with protein-specific antibody pairs as indicated. Cells were counterstained with α-BTX–A488 in green and DAPI in blue. Dashed lines indicate edges of cells. Bar: 25 µm.

Since both HDAC6 and paxillin accumulate at the NMJ (Fig. 5, A–D) and since paxillin was shown to inhibit the catalytic activity of HDAC6 (Deakin and Turner, 2014), we hypothesized that HDAC6 and paxillin interact in skeletal muscle cells. To test this, we performed coimmunoprecipitation assays (Fig. 5 E). Following paxillin immunoprecipitation with an anti-paxillin antibody, HDAC6 was detected by Western blot, with coimmunoprecipitated proteins demonstrating that HDAC6 can indeed bind to paxillin (Fig. 5 E). To confirm this interaction in vivo, we performed a proximity ligation assay (PLA) in dissociated TA muscle fibers. The presence of numerous PLA-positive spots throughout muscle fibers confirmed the interaction between HDAC6 and paxillin (see Fig. S3). This interaction was particularly visible at the NMJ (Fig. 5, F and G), whereas no staining was observed when control antibodies were used (Fig. 5, F and G). Altogether, coimmunoprecipitation and PLA experiments demonstrate that paxillin and HDAC6 interact in muscle cells, especially at the NMJ.

We further investigated if paxillin could regulate HDAC6 activity in skeletal muscle cells. We tested this by using either (a) a plasmid encoding shRNAs against paxillin (shPXN) or (b) a plasmid encoding paxillin fused with GFP (PXN-GFP). shPXN, PXN-GFP, shRNA-Control (pLKO), and GFP alone (the latter two being respective controls of shPXN and PXN-GFP) were individually transfected in C2C12 myoblasts (Fig. 5 H). The levels of acetylated tubulin in each condition were subsequently analyzed by Western blot (Fig. 5, I and K). These experiments showed that paxillin depletion promotes tubulin deacetylation (Fig. 5 J), whereas paxillin overexpression increases the levels of acetylated tubulin (Fig. 5 L). These data show that paxillin is a potent inhibitor of HDAC6 in muscle cells. The precise accumulation of paxillin under AChR-rich domains indicates that HDAC6 activity must be low in the vicinity of AChR clusters and high in paxillin-free areas.

### Formation of agrin-dependent AChR clusters is regulated by HDAC6 inhibition and paxillin

**Figure 6.**
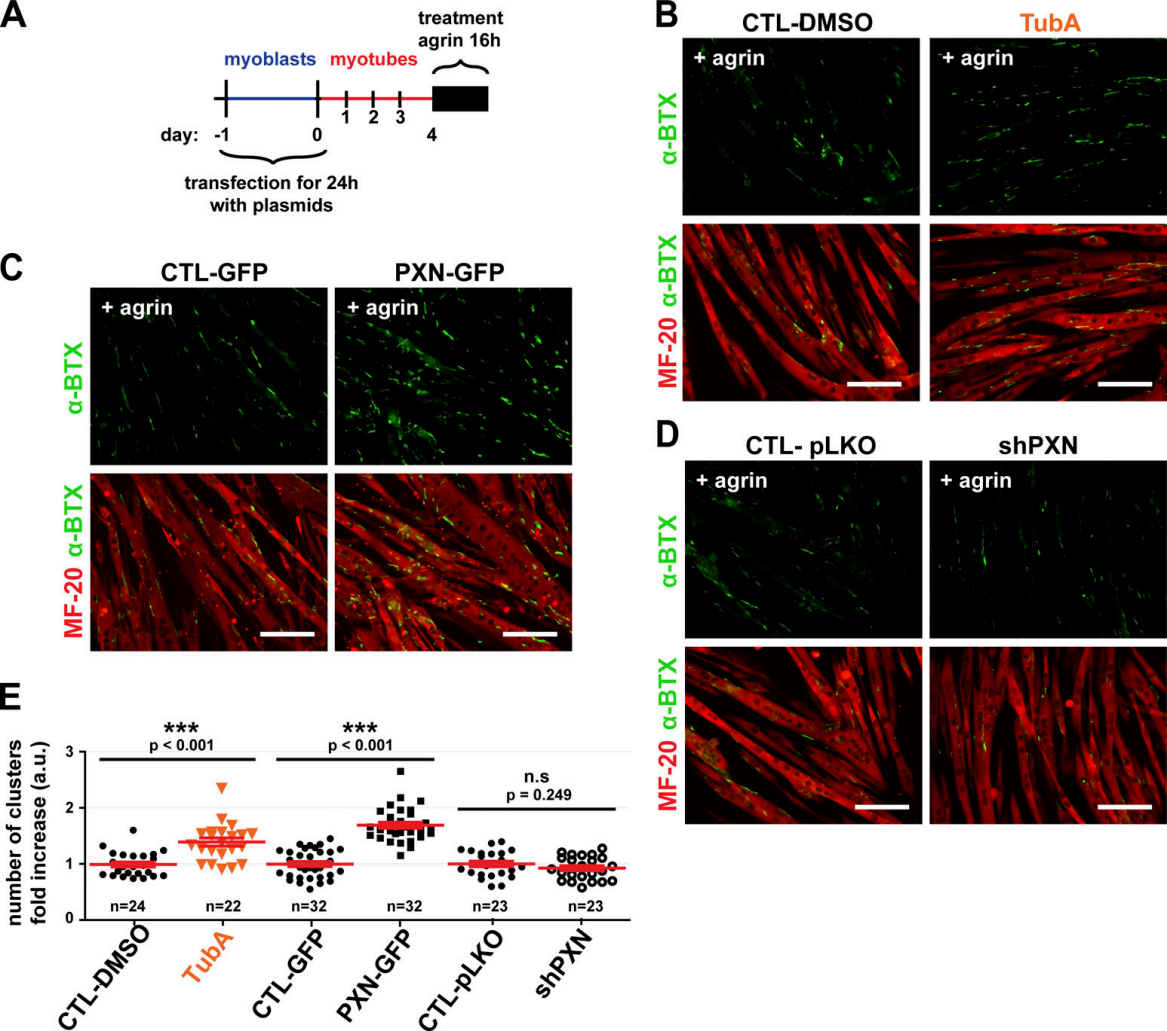
**The number of AChR clusters induced by agrin is dependent on levels of HDAC6 activation and paxillin expression.** **(A)** Schematic representation of the experimental time course. **(B–D)** After 4 d of differentiation, myotubes were treated with agrin (50 nM) for 16 h. **(B)** For HDAC6 inhibition conditions, WT myotubes were treated with either TubA (5 µM) or DMSO (CTL; 1 µl) at the same time as agrin. Myotubes were stained with MF-20 (in red). **(C and D)** Myoblasts were transfected as described in Fig. 5. For all conditions at day 5, AChRs clusters were labeled with α-BTX–A488 (in green) and myotubes with MF-20 (in red). **(E)** Quantifications of three independent experiments representing the total number of AChR clusters per field of view normalized to the area of all myotubes in each treated myotube condition compared with the respective control conditions (*n* = number of fields of 0.15 mm^2^ counted, between 22 and 32). Means ± SEM. ***, P < 0.001; n.s, not significant; Mann-Whitney *U* test. **(B–D)** Scale bars: 100 µm.

It is well established that both in vivo and in culture, formation of AChR clusters can be stimulated by agrin via its interaction with low-density lipoprotein receptor–related protein (LRP)‒4 and muscle-specific tyrosine kinase (MuSK; Gautam et al., 1996; DeChiara et al., 1996; Zhang et al., 2008; Kim et al., 2008). Therefore, we next evaluated whether HDAC6 inhibition with TubA or paxillin had an effect on the formation of AChR clusters induced by agrin. 4-d-old myotubes were treated with agrin for 16 h, and AChR molecules were labeled with α-BTX–A488 (Fig. 6 A). As expected, agrin induced the formation of AChR clusters (Fig. 6 B; CTL-DMSO). To examine the role of HDAC6, TubA was added concomitantly to agrin in the culture medium. The results show that inhibition of HDAC6 by TubA increased the number of AChR clusters by ∼40% (Fig. 6 B). In agreement with our results shown in Fig. 4, we conclude that MT acetylation via HDAC6 inhibition promotes the formation of AChR clusters.

To expand upon these findings, we modulated the expression of paxillin in myoblasts using PXN-GFP and shPXN as described in Fig. 5. Overexpression of PXN-GFP showed a ∼60% increase in the number of AChR clusters compared with the GFP control (Fig. 6, C and E). shPXN inhibited the expression of paxillin by ∼70% (Fig. 5, I and J) but only modestly reduced the differentiation index (∼25%; not shown) and did not alter the number of AChR clusters induced by agrin (Fig. 6, D and E). Together, these results further highlight the role of HDAC6 in AChR cluster formation and also indicate that regulation of MT acetylation via paxillin and HDAC6 provides a new signaling axis in the control of agrin-induced AChR insertion.

### Acetylated MTs achieved via HDAC6 inhibition regulate the structure of NMJs

**Figure 7.**
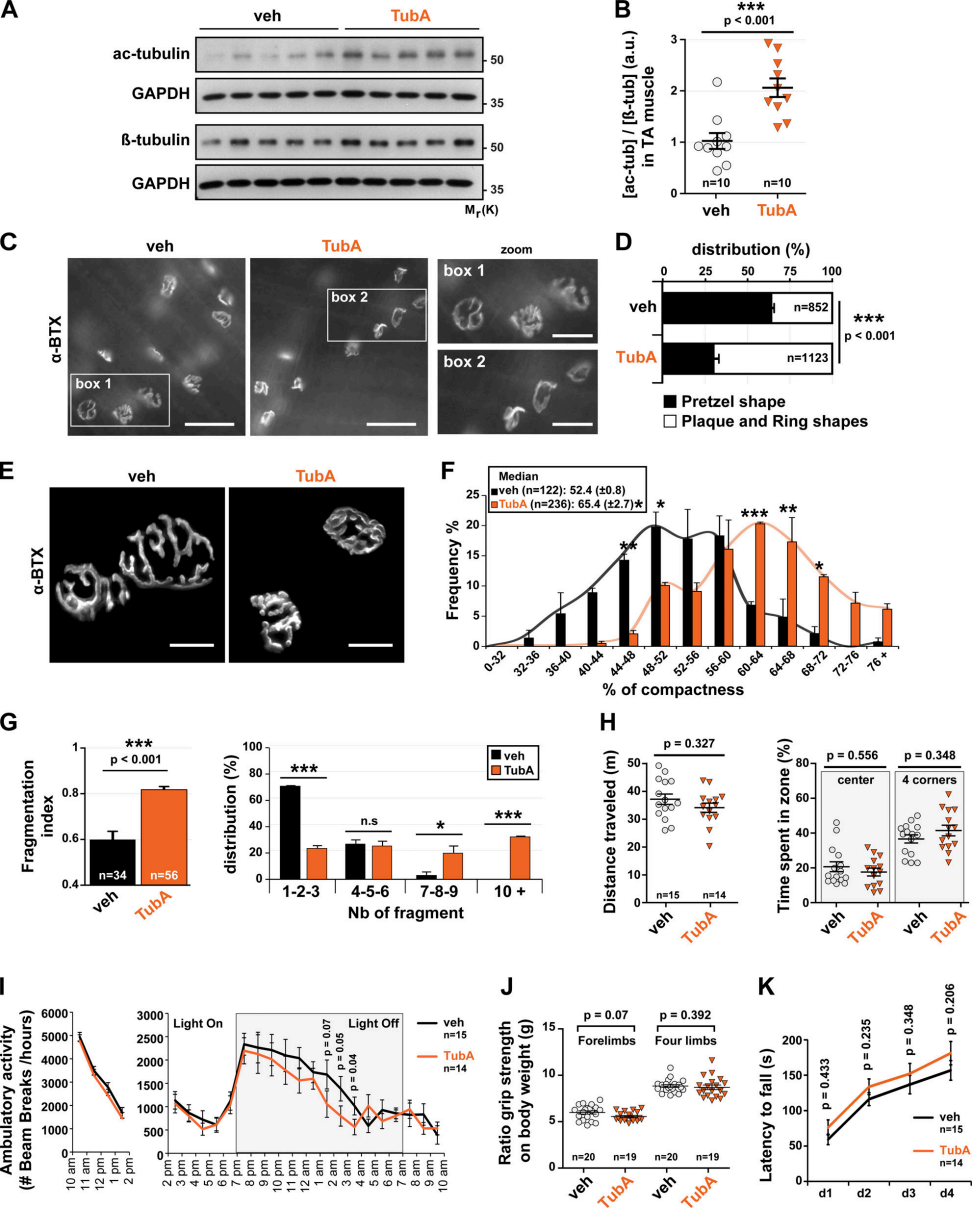
**In vivo, HDAC6 inhibition via TubA treatment regulated NMJ structure and did not affect behavioral disorders.** **(A and B)** 7-wk-old WT mice were treated with TubA or with vehicle-control (veh) for 31 consecutive days. To evaluate the level of α-tubulin acetylation in veh- and TubA-treated mice in TA muscles, Western blot analysis (A) and quantification (B) were performed. Quantification of acetylated tubulin protein level normalized to β-tubulin. GAPDH was used as a loading control (*n* = number of mice used per condition, 10). **(C)** Hemi-DIA muscle fibers were stained with α-BTX–A488 (in gray). **(D)** Total distribution between pretzel-like shape and plaque/ring shapes in hemi-DIA (*n* = total number of NMJs counted on five mice for each condition; veh = 852 and TubA = 1,123). **(E)** NMJs of isolated TA fibers labeled with α-BTX–A488 (in gray). **(F)** Graphical summary of NMJ compactness (*n* = total number of NMJs counted on five mice for each condition; veh = 122 and TubA = 236). **(G)** Fragmentation index and distribution of number of fragments have been quantified (*n* = total number of NMJs counted on three mice for each condition; veh = 34 and TubA = 56). **(H)** Open-field behavior. Distance traveled and time spent in the center or in corners of the open-field chamber are shown on the y axis. **(I)** Beam break test was realized for 12 h. Motor habituation and activity are shown on the y axis. **(J)** Grip strength was measured on a grid measuring maximal forelimb and hind limb grip strength normalized on body weight. **(K)** Rotarod test was performed on 4 d. Effect of TubA on the average time to fall off the rotarod. **(H, I, J, and K)** *n* = number of mice used per condition (veh = 15–20, and TubA = 14–19). Quantifications show means ± SEM. *, P < 0.05; **, P < 0.01; ***, P < 0.001; Mann-Whitney *U* test. Bars: 500 µm (C); 25 µm (E); inset magnifications (C; boxes 1, 2): 250 µm. n.s, not significant; β-tub, β-tubulin; Mr(K), relative molecular weight in kiloDalton; Nb, number; ac-tub and ac-tubulin, acetylated tubulin.

To assess the role of HDAC6 on MT acetylation at NMJs, we performed daily TubA intraperitoneal injections in 7-wk-old mice for 1 mo (25 mg/kg/d). Initially, we examined whether acetylated tubulin levels were increased in TA muscles of TubA- versus vehicle-treated mice. Western blots performed on TA muscles showed that the total relative amount of acetylated tubulin was indeed increased in TubA-treated mice by approximately twofold (Fig. 7, A and B) compared with vehicle-treated mice.

We next investigated the organization of NMJs in both diaphragm (DIA) and TA muscles. In a first set of experiments, hemi-DIA was collected and stained with α-BTX–A488 to label NMJs (Fig. 7 C). The morphology of NMJs in all hemi-DIA muscles was classified into two distinct categories (Bolliger et al., 2010): (a) “pretzel-like shape,” displaying a large number of invaginations and perforations and defined as mature NMJs, or (b) “plaque/ring shape,” which was categorized as abnormal NMJs. Representative images of each NMJ phenotype are shown in Fig. 7 C in zoom 1 and 2, respectively (Fig. 7 D). Detailed blinded morphological analyses revealed that the structure of NMJs was affected by the TubA treatment such that plaques or rings were clearly more abundant, representing ∼70% of all NMJs (P value <0.001 compared with vehicle). The ∼50% reduction in the percentage of classic pretzel-like–shaped NMJs in TubA-treated muscles highlights the impact of HDAC6 on the structure of NMJs.

**Figure S4.**
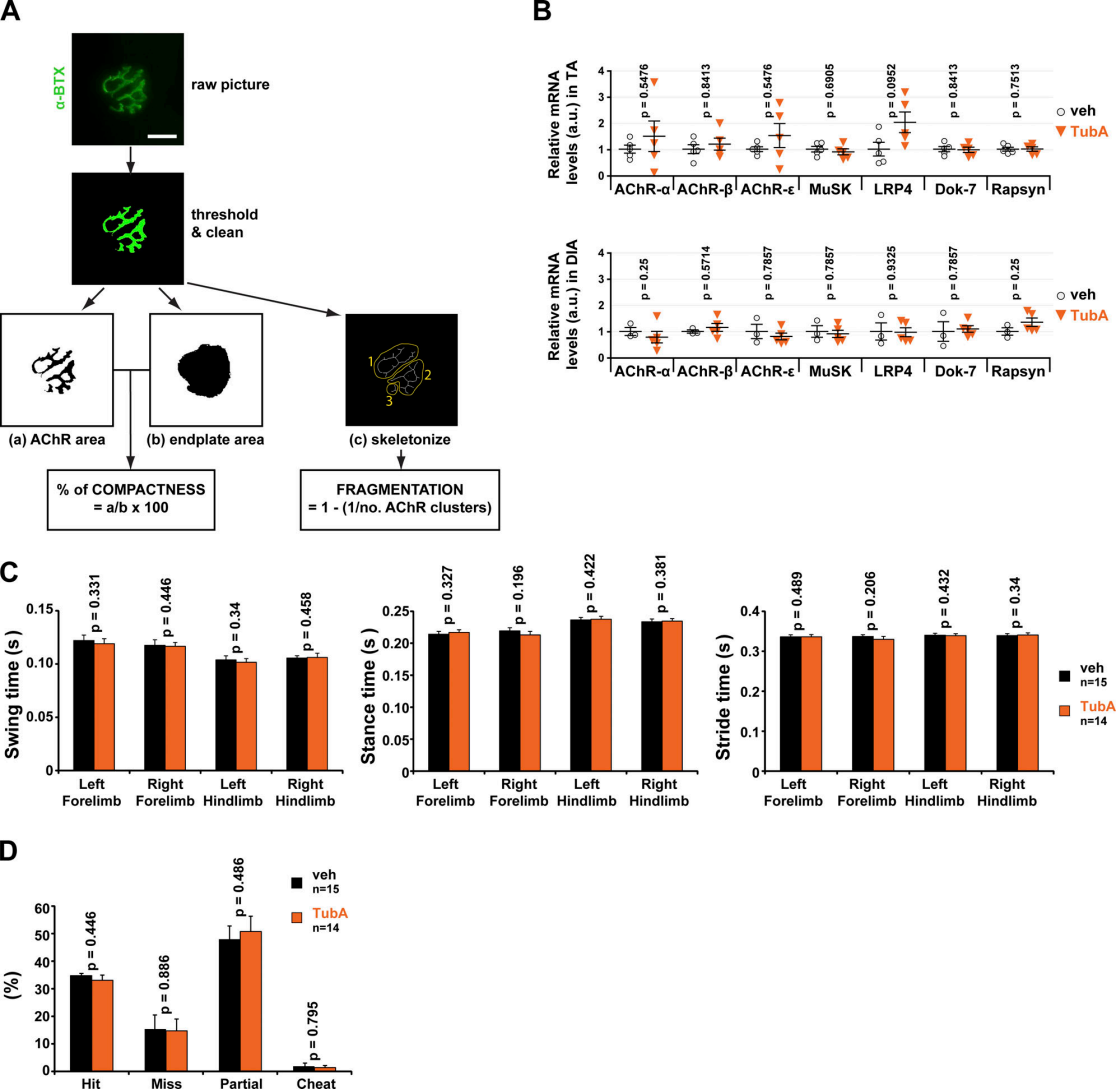
**Effects of tubA treatment on synaptic gene RNA levels and in vivo gait measurements.** **(A)** Overview of the NMJ-morph platform. Flowchart demonstrating the sequence of analyses for each NMJ using NMJ-morph (adapted from Jones et al., 2016). NMJs were stained with α-BTX–A488 (in green). Bar: 25 µm. **(B)** Endogenous relative levels of AChR α-subunit, AChR β-subunit, AChR ε-subunit, MuSK, LRP4, Dok-7, and rapsyn mRNA as determined by quantitative RT-PCR in TA and DIA muscles. Three reference genes (GAPDH, 18S, and actin) were used to normalize data. **(C)** DigiGait analysis. Gait measurements such as swing time, stance time, or stride time were performed. **(D)** Horizontal ladder. The numbers of successful steps (hit), slips/missed steps, partial steps, and cheat steps were quantified. *n* = number of mice used per condition (veh = 15, and TubA = 14). Graphs show means ± SEM. Mann-Whitney *U* test. veh, vehicle.

In a second series of experiments (Fig. 7 E), we performed a quantitative morphometric analysis of NMJs on isolated TA muscle fibers labeled with α-BTX–A488 (see Fig. S4 A, Materials and methods, and Jones et al., 2016). Results showed that in TA muscles from TubA-treated mice, NMJs were present in a more compacted and fragmented form than in vehicle-treated mice. The relative median NMJ compactness was increased from ∼50% in vehicle-treated mice to >65% in TubA-treated mice (P value = 0.02; Fig. 7 F). In addition, the index of NMJ fragmentation was increased by nearly 40% in TubA-treated mice compared with vehicle-treated mice (P value <0.001; Fig. 7 G). This indicates that HDAC6 inhibition increases the compactness and fragmentation of NMJs. These data are consistent with our data obtained with myotubes (see Fig. 4). Together, these data show that HDAC6 inhibition promotes a more compact and fragmented NMJ phenotype in vivo.

Lastly, we evaluated the effect of TubA on the expression of NMJ components by measuring relative mRNA levels of several synaptic genes coding for: AChR α-, β-, and ε-subunits, MuSK, LRP4, Dok-7, and rapsyn. No difference was observed in the levels of these seven synaptic mRNAs in both TA and DIA muscles from TubA- and vehicle-treated mice (Fig. S4 B). TubA therefore does not affect the expression of key synaptic genes.

### TubA-treated mice do not show behavioral abnormalities

To examine the functional impact of HDAC6 inhibition, TubA- or vehicle-treated mice were subjected to a series of blinded behavioral tests chosen to assess locomotion and motor properties. In open-field experiments, both TubA- and vehicle-treated mice traveled the same distance and displayed comparable levels of anxiety (Fig. 7 H). Interestingly, beam break counting indicated that TubA-treated mice showed a slight decrease in their nocturnal motor activity (Fig. 7 I). However, we did not observe any significant difference in the grip strength of both forepaws and all paws (Fig. 7 J). In addition, the TubA-treated mice did not show any significant difference in fatigue resistance as measured in rotarod experiment (Fig. 7 K), as well as in stepping/placing step or in their gait determined in DigiGait (Fig. S4 C) and horizontal ladder (Fig. S4 D) experiments. Altogether, these results indicate that TubA-treated mice had a motor performance similar to that of vehicle-treated mice, indicating that their NMJs remained functional despite the level of disorganization.

### The deletion of HDAC6 decreases AChR areas without affecting motor behavior

**Figure 8.**
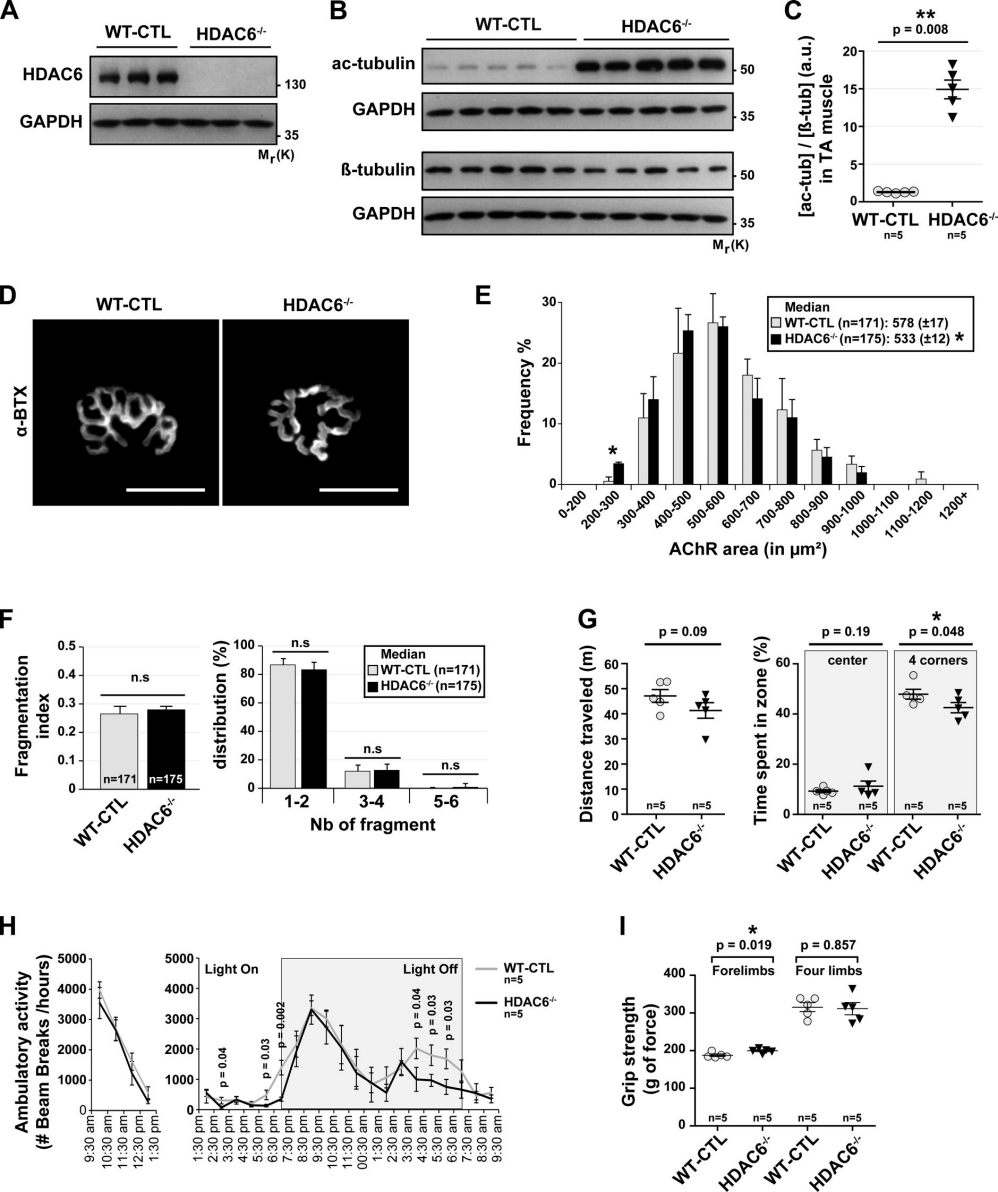
**In vivo CRISPR/Cas9 KO HDAC6 mice affect NMJs but do not lead in behavioral disorders.** 9-wk-old WT mice (WT-CTL) and KO HDAC6 mice (HDAC6^−/−^) were treated with TubA or with vehicle-control (veh) for 31 consecutive days. **(A)** To evaluate the level of HDAC6 expression, Western blots were performed. **(B)** Levels of α-tubulin acetylation in WT-CTL and HDAC6^−/−^ mice in TA muscles were evaluated by Western blot analysis. **(C) **Quantification of acetylated tubulin protein level normalized to β-tubulin. **(A and B)** GAPDH was used as a loading control (*n* = number of mice used per condition; WT-CTL = 5 and HDAC6^−/−^ = 5). **(D)** NMJs of isolated TA fibers labeled with α-BTX–A488 (in gray). **(E)** Graphical summary of NMJ compactness (*n* = total number of NMJs counted on five mice for each condition; WT-CTL = 171, and HDAC6^−/−^ = 175). **(F)** Fragmentation index and distribution of number of fragments have been quantified (*n* = total number of NMJs counted on five mice for each condition; WT-CTL = 171 and HDAC6^−/−^ = 175). **(G)** Open-field behavior. Distance traveled and time spent in the center or in corners of the open-field chamber are shown on the y axis. **(H)** Beam break test was realized for 12 h. Motor habituation and activity are shown on the y axis. **(I)** Grip strength was measured on a grid measuring maximal forelimb and hind limb grip strength. **(G–I)** *n* = number of mice used per condition (WT-CTL = 5, and HDAC6^−/−^ = 5). **(D)** Scale bars: 25 µm. n.s, not significant. β-tub, β-tubulin; Mr(K), relative molecular weight in kiloDalton; Nb, number; ac-tub and ac-tubulin, acetylated tubulin. Quantifications show means ± SEM. *, P < 0.05; **, P < 0.01; Mann-Whitney *U* test.

To further evaluate the role of HDAC6 on NMJ structure and function, we used CRISPR/cas9–generated HDAC6 knockout (KO) mice (HDAC6^−/−^ mice). As expected, no expression of HDAC6 was detected by Western blot in TA muscles using an anti-HDAC6 antibody (Fig. 8 A), thereby validating the mouse model and the specificity of the antibody. The absence of HDAC6 protein promoted the hyperacetylation of α-tubulin with a marked ∼15-fold increase compared with control mice (Fig. 8, B and C). The staining of dissociated TA fibers with α-BTX–A488 (Fig. 8 D) showed that the relative median AChR area was decreased in HDAC6^−/−^ compared with WT mice used as control (WT-CTL; Fig. 8 E). However, the fragmentation index and the number of fragments were not significantly affected in KO mice (Fig. 8 F).

To evaluate the functional properties of NMJs in HDAC6^−/−^ mice, we performed a series of behavioral tests, as described in Fig. 7. Our results show that motor function was not affected in HDAC6^−/−^ mice in open-field experiments (Fig. 8 G, distance traveled). HDAC6^−/−^ mice spent slightly less time in corners (Fig. 8 G, time spent in zone), indicating no major difference in anxiety. As with TubA-treated mice in Fig. 7 I, HDAC6^−/−^ mice displayed a lower nocturnal motor activity in beam break experiments (Fig. 8 H). In contrast, the maximal forelimb peak force was modestly increased in HDAC6^−/−^ mice, while no changes were observed for the hind limbs (Fig. 8 I). Altogether, these data demonstrate that the deletion of HDAC6 by CRISPR/cas9 in vivo decreased AChR area without major impact on KO mouse motor behavior.

### Lysine 40 of α-tubulin and catalytic domains of HDAC6 control the structure of NMJs

**Figure 9.**
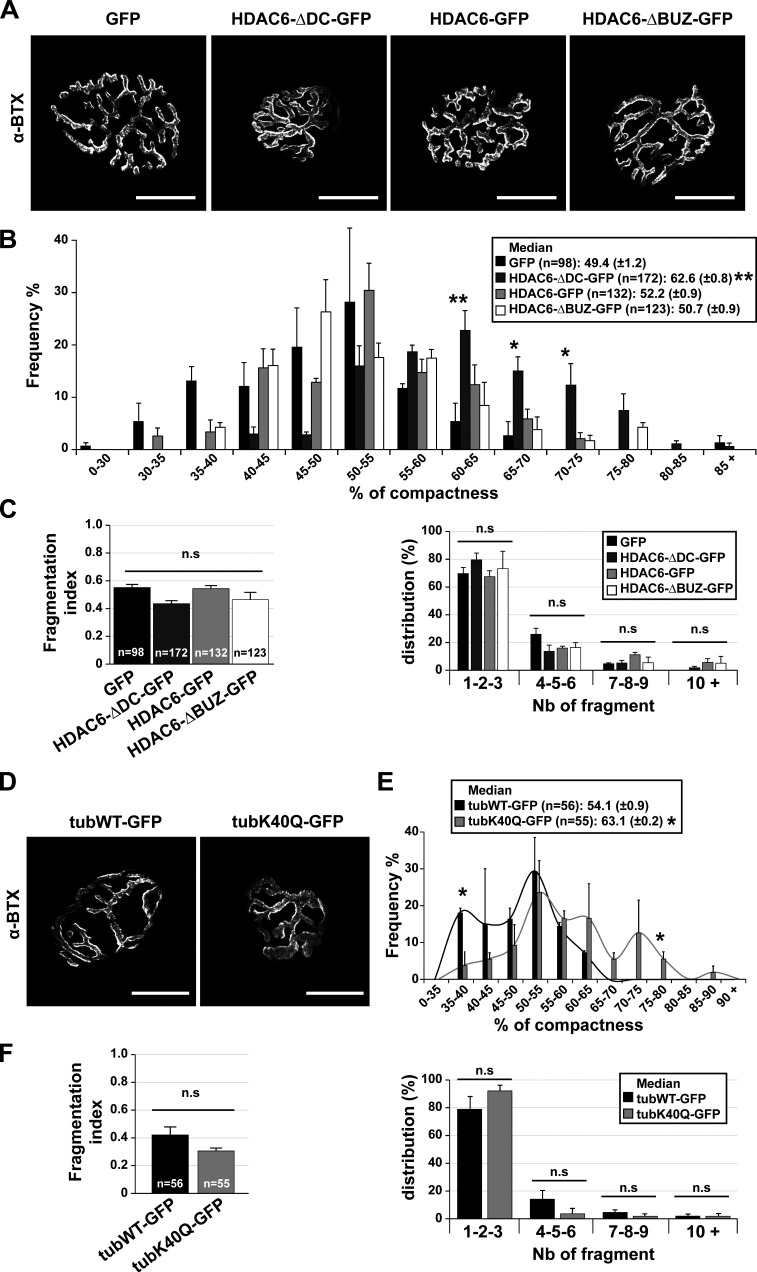
**In vivo, NMJ structure is regulated by HDAC6 inhibition via its catalytic domains and its interaction with lysine 40 of α-tubulin.** **(A and D)** TA fibers were coelectroporated for 7 d with either one HDAC6 mutant (HDAC6-GFP; HDAC6-ΔDC-GFP; HDAC6-ΔBUZ-GFP) or a control GFP (A) or with WT tubulin (TubWT-GFP) or a mutant (TubK40Q-GFP; D). Myofibers were labeled with α-BTX–A594 (in gray), and only GFP-positive myofibers were selected. **(B and E)** Graphical summary of NMJ compactness (*n* = total number of NMJs, three to five mice for each condition; B and C between 98 and 172; E and F, TubWT-GFP = 56 and TubK40Q-GFP = 55). **(C and F)** Fragmentation index and distribution of number of fragments have been quantified (*n* = total number of NMJs counted on three to five mice for each condition). Graphs show means ± SEM. *, P < 0.05; **, P < 0.01; n.s, not significant; Mann-Whitney *U* test. **(A and D)** Bars: 25 µm. n.s, not significant; Nb, number.

**Figure S5.**
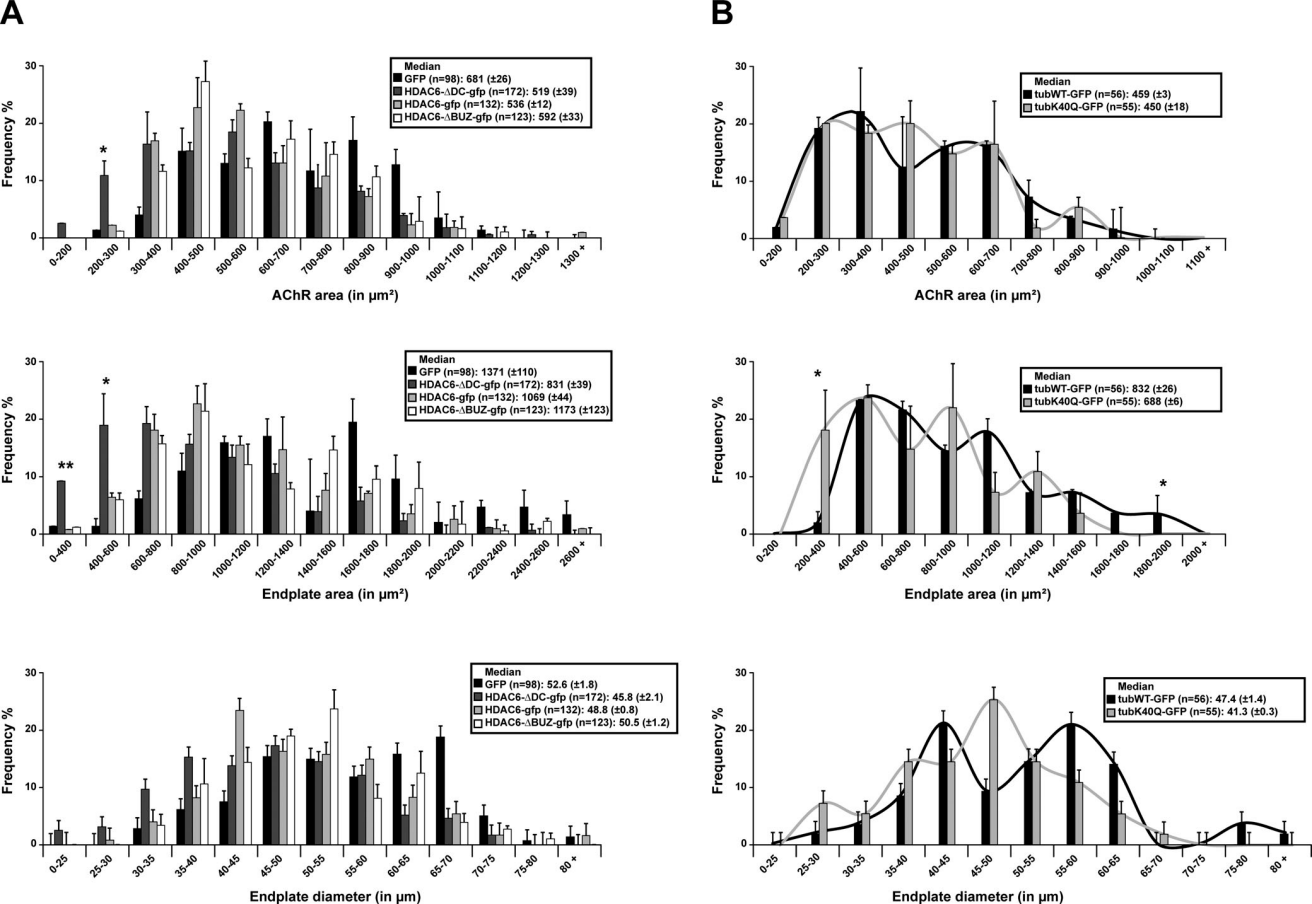
**AChR area, endplate area, and diameter distribution in mice electroporated.** **(A and B)** AChR area, endplate area, and diameter distribution in mice electroporated with either mutants of HDAC6 (A) or mutants of tubulin (B). Graphical summary of NMJ AChR area, endplate area, and diameter (*n* = total number of NMJs counted on five mice for each condition). Graphs show means ± SEM. *, P < 0.05; **, P < 0.01; Mann-Whitney *U* test.

To further examine the influence of MT acetylation on NMJ structure, HDAC6 mutants were electroporated in TA muscle fibers of WT mice (Fig. 9 A). Different constructs were employed: (a) a WT HDAC6 fused with GFP (HDAC6-GFP); (b) a mutant of HDAC6 deprived of its catalytic domains (HDAC6-ΔDC-GFP); and (c) a mutant of HDAC6 deprived of its ubiquitin-binding domain (used as a control; HDAC6-ΔBUZ-GFP). To express these mutants in TA muscles, injections of plasmid DNA followed by electroporation were performed as previously described (Ravel-Chapuis et al., 2007). 7 d after electroporation, myofibers were dissected and stained with α-BTX–A594. The compactness and the fragmentation of each NMJ were analyzed (Fig. 9, B and C). Under control conditions (GFP, HDAC6-GFP, and HDAC6-ΔBUZ-GFP), we did not detect any differences in the relative median NMJ compactness (∼50%; P value = 0.234 compared with GFP). In contrast and in agreement with the results obtained with TubA treatments (Fig. 7, E and F), muscle fibers electroporated with HDAC6-ΔDC-GFP had more compacted NMJs (median ∼63%; P value = 0.003 compared with GFP; Fig. 9 B and Fig. S5 A) compared with control conditions, indicating that this mutant acts as dominant-negative. However, neither the fragmentation index nor the distribution of the number of fragments was affected (Fig. 9 C). Together, these results indicate that the catalytic domain of HDAC6, but not the ubiquitin-binding domain, is essential for the effect of HDAC6 on the structure of NMJs.

Finally, to better define the role of acetylated tubulin and the implication of the MT network on the structure of NMJs, we used mutants of α-tubulin. We used an α-tubulin mutant fused with GFP, in which lysine 40 was substituted with glutamine (TubK40Q-GFP), to mimic acetylated tubulin (Fig. 9 D). WT tubulin fused with GFP (TubWT-GFP; used as control) and TubK40Q-GFP were injected and electroporated in TA muscles of WT mice. 1 wk later, staining of dissociated TA fibers from TubK40Q-GFP–injected mice with α-BTX–A594 revealed that the relative median NMJ compactness was ∼63% (P value = 0.021 compared with TubWT-GFP), whereas in the TubWT-GFP condition, this median was ∼54% (Fig. 9 E and Fig. S5 B). As observed with overexpression of HDA6 mutants, the fragmentation index and the distribution of the number of fragments from TubK40Q-GFP were not changed compared with the control (Fig. 9 F). Collectively, these data show that tubulin acetylation at lysine 40 and HDAC6 catalytic activity modulate NMJs’s architecture.

## Discussion

**Figure 10.**
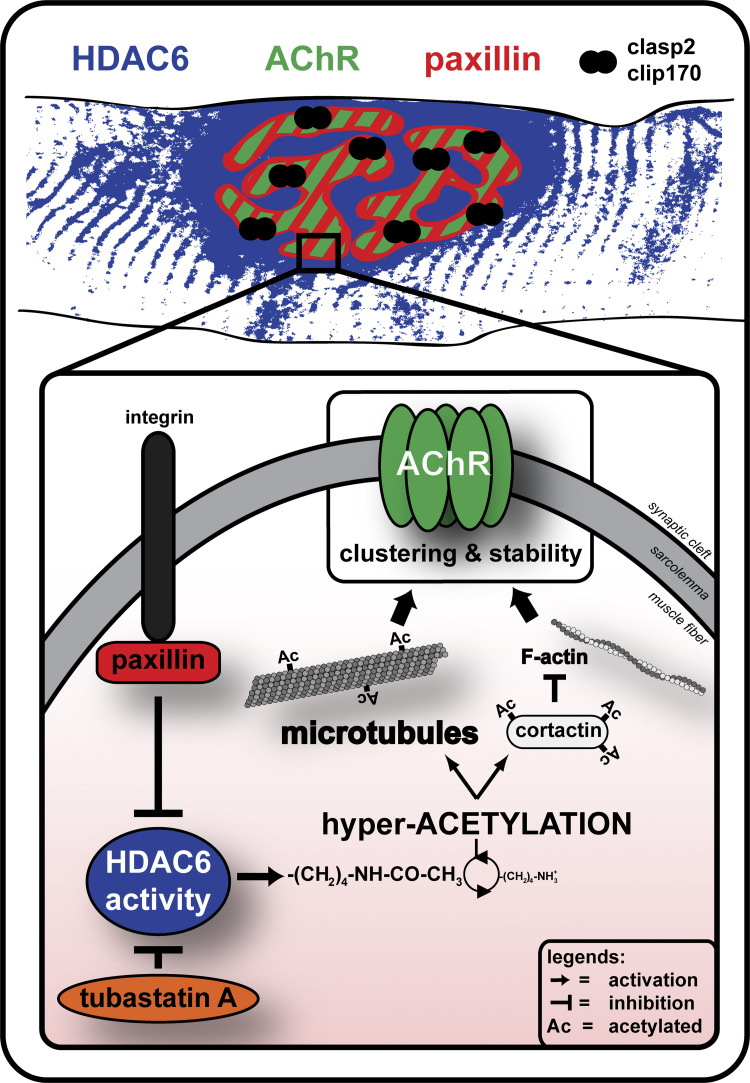
**Proposed model of MT/HDAC6/paxillin axis at the NMJ.** HDAC6 inhibitors such as TubA and overexpression of paxillin induce a decrease in HDAC6 activity that leads to a hyperacetylation of tubulin. This tubulin hyperacetylation plays a role in the clustering and stability of AChRs at the NMJ. While paxillin colocalizes perfectly with AChRs, HDAC6 is enriched but only partially accumulates at the NMJ. We propose that at the NMJ, HDAC6 is specifically inhibited at sites of AChR incorporation by its endogenous inhibitor paxillin.

Although previous work has shown the presence of a stable network of MTs at NMJs (Jasmin et al., 1990; Schmidt et al., 2012), the functional relevance of this specialized network of MTs has yet to be fully elucidated. In the present study, we specifically addressed this question and also examined the role of HDAC6 in controlling the organization of the postsynaptic MT network as well as the structure of NMJs. By targeting the main tubulin deacetylase, we showed that MT acetylation controls the dynamics and distribution of AChRs in skeletal muscle fibers and cultured myotubes. Indeed, HDAC6 inhibition protects against MT disorganization and markedly influences the structure of NMJs. Furthermore, we report that the endogenous HDAC6 inhibitor paxillin accumulates at NMJs where it colocalizes with AChR aggregates. Altogether, our results indicate that stable MTs at NMJs contribute to the focal insertion of AChRs into the postsynaptic membrane. Moreover, these findings provide new and key insights into how a synaptic MT/HDAC6/paxillin axis controls the structure of NMJs, thereby ultimately regulating the accumulation of AChRs at postsynaptic sites (Fig. 10).

### HDAC6 is a new component of NMJs

Earlier studies showed that tubulin is highly acetylated at chick NMJs, indicating the presence of large amounts of stable MTs at the neuromuscular synapse (Jasmin et al., 1990). Here, we confirmed that at mammalian NMJs, the MT network is dense and rich in stable MTs as evidenced by the presence of high levels of tubulin acetylation. Our data further indicate that MT acetylation controls the distribution of AChRs at postsynaptic membranes in culture and in vivo, a result in excellent agreement with previous findings that examined the effects of MT-disrupting drugs on the stability, formation, and removal of AChRs (Connolly, 1984; Prives et al., 1982). In mammals, the major enzymes controlling tubulin acetylation are α-TAT1 and HDAC6 (Kalebic et al., 2013; Hubbert et al., 2002). Here, we further report that conversely to α-TAT1, HDAC6 is highly enriched within the postsynaptic domain of NMJs, consistent with the idea that it participates in the organization of the postsynaptic apparatus. Altogether, these findings highlight the important functional role of stable MTs at NMJs, emphasizing also the key roles of HDAC6 and paxillin in regulating the acetylation status of tubulin and hence the stability of the postsynaptic MT network.

### Increased acetylation of tubulin via HDAC6 inhibition promotes MT stability without altering mouse motor behavior

HDAC6 is an unusual HDAC harboring two catalytic domains. HDAC6 is cytoplasmic, and its major substrate is lysine 40 of α-tubulin. Tubulin acetylation occurs on polymerized MTs and affects MT dynamics and stability by slowing down the rate of MT growth and shrinkage (Janke and Bulinski, 2011). Moreover, long-lived MTs are lost in the absence of tubulin K40 acetylation, and tubulin acetylation at K40 protects MTs against stress-induced material fatigue because acetylation directly alters the mechanical properties of MTs, making them more flexible and less susceptible to breakage (Xu et al., 2017; Portran et al., 2017). MTs containing mutant K40Q α-tubulin, which mimics acetylated tubulin, are more resistant to cold-induced disassembly, whereas MTs containing mutant K40R α-tubulin, which mimics nonacetylated tubulin, are less resistant to cold (Mao et al., 2017). In *Drosophila*, treatment with HDAC6 inhibitors increases tubulin acetylation and preserves MTs from deterioration (Haggarty et al., 2003; Kaluza et al., 2011; Xiong et al., 2013). In the present study, we used TubA to inhibit HDAC6 because of its selectivity for HDAC6 (IC50 at 0.015 µM ± 0.001; Butler et al., 2010). Consistent with previous work (Zilberman et al., 2009; Mao et al., 2017; Xiong et al., 2013), our results show that in skeletal muscle cells, stimulation of MT acetylation via HDAC6 inhibition protects MT networks. Indeed, our results show that treatment of muscle with the HDAC6 inhibitor TubA is capable of maintaining the organization of MT networks against cold- or nocodazole-induced MT depolymerization.

Unlike with other histone deacetylases, inhibition of HDAC6 does not appear to be associated with any serious toxicity, making it an excellent drug target (Witt et al., 2009). Most of the selective HDAC6 inhibitors, such as ACY-1215 or TubA, are able to cross the blood–brain barrier (Cook et al., 2014; Butler et al., 2010; Zhang et al., 2014b; Selenica et al., 2014; Zwick et al., 2016). Our results show that daily injection of TubA in mice for 1 mo did not affect the behavior of mice, as reflected by levels of anxiety, motor function, motor activity and habituation, motor behavior and coordination, fatigue resistance, stepping, and gait of rodents. In these mouse muscles, expression of synaptic genes such as MuSK, LRP4, Dock-7, rapsyn, or AChR α-, β-, and ε-subunits was not altered by HDAC6 inhibition. Indeed, it was already reported that TubA rescues memory function in Alzheimer’s disease transgenic mice without significant effects on anxiety levels (Zhang et al., 2014b) and rescues hyperactivity phenotype and memory impairment in mouse models of tau deposition (Selenica et al., 2014). Similar to the experiments performed with TubA, mice with an absence of HDAC6 protein present normal anxiety, motor function, motor activity, and habituation. HDAC6^−^/^−^ mice are viable, fertile, and live normally (Zhang et al., 2008). Together, these results highlight that no overt behavioral defects are present in mice depleted of HDAC6 protein or treated with a specific inhibitor of catalytic activity domains of HDAC6.

### HDAC6 inhibition affects AChR clustering

In our experiments, TubA did not affect HDAC6 localization at NMJs, suggesting that the catalytic activity of HDAC6 is not essential for its subcellular localization. Conversely, TubA treatment markedly affected the distribution of AChR clusters. In cultured muscle cells, TubA reduced the area of individual AChR clusters while increasing their total number. In vivo, TubA increased the occurrence of abnormal NMJs in both DIA and TA muscles as reflected by increased compactness and fragmentation. Slater (2019) proposed a model in which NMJ “reorganization” is the outcome of a normal process by which the NMJ is maintained in an effective state. NMJ reorganization would therefore be a form of regeneration, rather than a degeneration (Slater, 2019). Our results are in agreement with this model and with work from others showing that MT acetylation protects *Drosophila* NMJs from alterations (Mao et al., 2017).

At synapses, the density of neurotransmitter receptors in the postsynaptic membrane is determined by the balance between receptor insertion and removal. It is currently widely accepted that receptor accumulation at postsynaptic sites involves aggregation of receptors by protein–protein interactions and focal targeting of receptors to the postsynaptic membrane by specialized actin and MT networks. However, the role of MT dynamics in synaptic function is only beginning to emerge. Indeed, it was only recently reported that dynamic MTs enter dendritic spines transiently in a way that appears to be dependent on synaptic activity and that correlates with long-term potentiation (Hu et al., 2008; Jaworski et al., 2009; Gu et al., 2008). In a study on AChR cluster formation, agrin was previously shown to capture MTs at sites of AChR clustering via the MT-binding protein CLASP2 (Schmidt et al., 2012). Altogether, our results indicate that in muscle in vivo, TubA, HDAC6 KO, HDAC6 inactive mutant, and tubulin mutant mimicking acetylation all induce remodeling of NMJ structure without affecting expression of synaptic mRNAs. This indicates that promoting tubulin acetylation, and therefore stabilization of the MT network at NMJ, affects the distribution of AChRs.

### Tubulin acetylation via inhibition of HDAC6 increases AChR cluster insertion in cultured myotubes

Previous studies have shown that in cultured myotubes, AChR clusters have a half-life of 5–24 h depending on cell types and experimental conditions (Devreotes and Fambrough, 1975; Trinidad and Cohen, 2004; Wang et al., 1999; Kim and Nelson, 2000; Bruneau et al., 2005b). In our conditions, we observed that AChR clusters had a half-life of ∼10 h. Furthermore, we show here that MT depolymerization by nocodazole increased the half-life of AChR clusters. Conversely, MT stabilization with taxol, TSA, or TubA decreased the half-life of AChR clusters. These findings imply that either the turnover or the distribution (targeting) of AChRs is affected by MT stability. In control conditions, two thirds of new receptors incorporated were targeted directly to preexisting clusters. This result is consistent with previous studies that showed that a significant number of newly inserted receptors are targeted directly to existing clusters (Bursztajn et al., 1985). Interestingly, increasing MT stability and/or acetylation significantly increased the rate of AChR removal from clusters while concomitantly also increasing the insertion of AChR into novel areas of the sarcolemma. Naturally, this reduces the half-life of AChR clusters. Altogether, our results therefore indicate that acetylated MTs and HDAC6 play a key role in the membrane insertion and removal of AChRs in myotubes.

HDAC6 mainly deacetylates tubulin but has many other substrates such as cortactin (Zhang et al., 2007). Originally identified as a substrate of the Src tyrosine kinase, cortactin plays an important role in regulating cell motility. It interacts with F-actin to promote polymerization and branching. F-actin and focal adhesion proteins act to stabilize the relative positions of the postsynaptic membrane, the subsynaptic nuclei, and the sarcomeres during contraction (Yorifuji and Hirokawa, 1989; Sealock et al., 1989). F-actin and several proteins that bind to it, such as cortactin, are enriched at the NMJ and at AChR clusters in muscle cells. Indeed F-actin is thought to be involved in anchoring AChRs along with other proteins involved in NMJ formation and maintenance (Dai et al., 2000; Borges and Ferns, 2001). Inhibition of actin polymerization blocks the aggregation of AChRs in response to synaptogenic stimulation (Dai et al., 2000; Madhavan and Peng, 2003). Cortactin was found at areas of dynamic actin assembly, such as at the leading edge of migrating cells (e.g., in membrane ruffles; Wu and Parsons, 1993). It has been described that acetylation of cortactin prevents its localization to membrane ruffles, inhibits cell motility, and reduces its interaction with F-actin (Zhang et al., 2007). This reduction of interaction between cortactin and F-actin elicits a diminution in tyrosine phosphorylation of cortactin and leads to an inhibition of agrin-induced AChR clustering in myotubes (Madhavan et al., 2009). Altogether, previous work on cortactin suggests that HDAC6 function at the NMJ could also involve F-actin/cortactin regulation. In the future, it will be interesting to investigate this possibility and how this signaling pathway cooperates with paxillin/HDAC6 (see below) to potentially modulate the organization of AChR clusters.

### The Paxillin/HDAC6 axis is important for agrin-dependent AChR cluster formation

Paxillin was initially shown to control cell adhesion and migration through its ability to localize to sites of focal adhesion (Brown and Turner, 2004; Deakin and Turner, 2008, 2011). Recently, the mode of action of paxillin at adhesion sites was unraveled with the finding that paxillin regulates MT acetylation and stability by inhibiting HDAC6 deacetylase activity (Deakin and Turner, 2014). Here, we show that paxillin and HDAC6 are associated in muscle cells and that this association is present at NMJs. Our results further show that paxillin depletion in muscle cells induces MT hypoacetylation, whereas paxillin overexpression increases MT acetylation.

To investigate the possible involvement of paxillin in regulating AChR clustering, we examined its localization at individual NMJs by immunofluorescence. Our data show that paxillin precisely colocalizes with AChR molecules. To determine whether paxillin influences AChR recruitment, we examined the effect of paxillin inhibition, depletion, or overexpression on AChR cluster formation in response to agrin. Altogether, our results show that paxillin favors AChR cluster formation in response to agrin.

HDAC6 accumulates at NMJs with a broader distribution than AChRs. Conversely, paxillin appears to tightly colocalize with AChRs. Since paxillin is a known inhibitor of HDAC6, this suggests that HDAC6 is specifically inhibited at sites of AChR incorporation. This correlates with the observation that HDAC6 inhibition promotes MT acetylation and formation of AChR clusters. The fact that HDAC6 was specifically enriched at the NMJ and that paxillin was not reported to regulate α-TAT1 argues in favor of a prominent role of HDAC6 rather than α-TAT1 in the regulation of tubulin acetylation at the NMJ. In addition, α-TAT1 KO muscles do not show obvious perturbations of NMJ structure (unpublished results).

Altogether, these findings indicate that the differential distribution of HDAC6 and paxillin at NMJs generates a fine balance of nonacetylated and acetylated MTs, the later colocalizing with paxillin and AChRs. Such an MT interplay generates cold and hot spots for AChR insertion at the membrane, thus contributing to generation of the highly focused postsynaptic distribution of AChRs. Our results thus support a model in which, similar to what occurs at focal adhesion sites, local inhibition of HDAC6 by paxillin generates bundles of stable acetylated MTs that favor anterograde and retrograde protein trafficking toward primary gutters of NMJs (Fig. 10).

## Materials and methods

### Ethics statement

Procedures using animals were approved by the University of Ottawa Animal Care Committee and were in compliance with the guidelines of the Canadian Council on Animal Care and the Animals for Research Act. Procedures were also performed in accordance with French and European legislations on animal experimentation.

### Antibodies, staining, and drugs

All primary antibodies used in this study are presented in Table 1. Secondary antibodies used for immunofluorescence studies were coupled to Alexa-Fluor 488 or Alexa-Fluor 546 (Molecular Probes) or to Cy3 or Cy5 (Jackson ImmunoResearch Laboratories). Secondary antibodies used for Western blotting were either HRP coupled anti–rabbit-IgG polyclonal antibodies (Jackson ImmunoResearch Laboratories) or HRP goat anti–mouse-IgG antibodies (Millipore). To visualize the NMJ for immunofluorescence studies, we used α-BTX at 5 µg/ml conjugates with either Alexa-Fluor 488 or Alexa-Fluor 594. All drugs used in this study are presented in Table 2.

**Table 1.** Antibodies

**Antibody name**	**Type**	**Dilution**		**Provider**
		**Immunostaining**	**Western blotting**	
βTub 2.1 (tubulin)	Mouse monoclonal	1:500	1:10,000	Sigma, clone TUB2.1, #T5201
αTubulin (11H10)	Rabbit monoclonal	1:100	1:1,000	Cell Signaling, #2125
Acetylated tubulin	Mouse monoclonal	1:200	1:2,000	Sigma, clone 6-11 B-1, #T7451
HDAC6	Rabbit polyclonal		1:4,000	Sigma, #SAB4500011
HDAC6 (D21B10)	Rabbit monoclonal	1:400	1:4,000	Cell Signaling, #7612
Paxillin (Y113)	Rabbit monoclonal	1:500	1:10,000	Abcam, #ab32084
Paxillin (5H11)	Mouse monoclonal	1:100		Invitrogen, #MA5-13356
Myosin Heavy Chain sarcomere	Mouse monoclonal	1:200		DSHB, #MF20
Normal mouse IgG	Mouse monoclonal	1:100		DSHB, #MF20
GFP	Mouse monoclonal		1:1,000	Roche, #11 814 460 001
GAPDH (HRP)	Goat polyclonal		1:20,000	Abcam, #ab85760
C6orf134 (tubulin acetyltransferase)	Rabbit polyclonal	1:200	1:2,000	Abcam, #ab58742

**Table 2.** Drugs

**Drug name**	**Synonym**	**Target**	**Concentration**	**Provider**
TubA	TubA	HDAC6	5 µM	APExBIO, #A4101
TBC	TBC	HDAC6	5 µM	Sigma, #SML0065
N-hydroxy-4-(2-[(2-hydroxyethyl)(phenyl)amino]-2-oxoethyl)benzamide	HPOB	HDAC6	5 µM	APExBIO, #B4890
Rocilinostat	ACY-1215	HDAC6	0.5 µM	APExBIO, #A4083
Trichostatin A	TSA	Pan-HDACs	0.1 µM	Calbiochem, #647
Paclitaxel	taxol	MT network	10 µM	Sigma, #T7402
Nocodazole	noco	MT network	10 µM	Sigma, #M1404

All drugs were solubilized in DMSO.

### Plasmids and shRNA

For C2C12 transfection and muscle fiber electroporation experiments, paxillin-GFP (#15233), HDAC6-GFP (#36188), HDAC6-ΔDC-GFP (#36189), HDAC6-ΔBUZ (#36190), TubWT-GFP (#64060) and TubK40Q-GFP (#32912) plasmids were obtained from Addgene. For knockdown of paxillin in C2C12 cells, shPXN plasmid came from Open Biosystems, GE Dharmacon RMM3981-201801357 (Clone ID: TRCN0000097194).

### Animal models, treatments, and preparation of muscle

Control C57 black 10 (C57b10; Charles River) male mice were used in this study. HDAC6^−^/^−^ mice were provided by Jackson Laboratory (C57BL/6J-Hdac6^em2Lutzy^/J and C57BL/6J male mice). C57b10 WT mice were treated for 31 consecutive days with either TubA (APExBIO, #A4101; 25 mg/kg/d, intraperitoneally) solubilized in 2% DMSO or saline supplemented with 2% DMSO (vehicle-control; d’Ydewalle et al., 2011; Ota et al., 2016). After treatment, muscles were dissected and (a) frozen and crushed in liquid nitrogen for protein and RNA extraction; (b) embedded in Tissue-Tek OCT compound (VWR) and frozen in isopentane cooled with liquid nitrogen for cryostat sectioning; or (c) manually dissociated. Muscle fiber dissociation (Osseni et al., 2016) was performed with the following modifications: single TA fibers were fixed 30 min at room temperature in PBS–4% paraformaldehyde permeabilized 60 min in PBS–1% Triton X-100 at 30°C before saturation and incubation with antibodies, as described below.

### Cell culture and transfection

C2C12 cells were seeded on Matrigel-coated (Matrigel matrix; Corning) 35-mm-diameter plates and were maintained as myoblasts in DMEM supplemented with 10% fetal bovine serum and 1% penicillin-streptomycin (Multicell). Then cells were differentiated in differentiation medium (DMEM medium supplemented with 2% horse serum; Bio-Media). Cells grown in 35-mm-diameter plates were treated for either Western blot or immunofluorescence. For Western blot, cells were collected by trypsinization, washed with PBS, centrifuged, and stored at −20°C until used. For immunofluorescence, cells were fixed for 20 min in PBS–4% paraformaldehyde at room temperature, washed in PBS, and stored at 4°C until used. Transient transfections were performed using Lipofectamin Plus Transfection reagent (Invitrogen), according to the manufacturer’s protocol.

### RNA extraction, reverse transcription, and PCR

Total RNA was extracted from TA muscles using TriPure isolation reagent (Roche Diagnostics) per the manufacturer’s recommendations. TriPure-extracted RNA was treated for 60 min with DNase I (Invitrogen) to eliminate possible genomic DNA contamination. Real-time quantitative PCR was performed on reverse-transcribed RNA using the QuantiTect SYBR Green PCR kit (Qiagen) on an MX3005p real-time PCR system (Stratagene). Primers were designed using Primer 3 software from gene sequences obtained from GenBank. Primer specificity was determined using a BLAST search. Oligonucleotide primers used for PCR analysis were as follows: AChRα, forward: 5′-ACC​TGG​ACC​TAT​GAC​GGC​TCT-3′, reverse: 5′-AGT​TAC​TCA​GGT​CGG​GCT​GG-3′; AChRβ, forward: 5′-CAT​CAT​CGC​TCA​CCC​CAC-3′, reverse: 5′-ACG​GTC​CAC​AAC​CAT​GGC-3′; AChRε, forward: 5′-GCG​GAG​CGA​ACT​CGT​GTT​TG-3′, reverse: 5′-ACA​GCA​GCG​GAT​TTC​TGG​GG-3′; MuSK, forward: 5′-CTT​CAG​CGG​GAC​TGA​GAA​AC-3′, reverse: 5′-TGT​CTT​CCA​CGC​TCA​GAA​TG-3′; LRP4, forward: 5′-ACC​AGG​AAA​TCA​TTC​GCA​ACA​AGC-3′, reverse: 5′-TGG​GGC​AGG​CAC​AGG​TGT​AGT​TCT​G-3′; Dok-7, forward: 5′-GCA​CAG​GTT​CCA​TGT​GAC​AG-3′, reverse: 5′-CTC​ATC​TGC​TCT​CCC​TCA​GC-3′; and Rapsyn, forward: 5′-GAC​TAT​GGC​AAA​GGC​TGG​AG-3′, reverse: 5′-CAG​GGC​AAT​CTT​CAT​GGA​CT-3′. The data were normalized to β-actin, forward: 5′-CCC​TGT​ATG​CCT​CTG​GTC​GT-3′, reverse: 5′-ATG​GCG​TGA​GGG​AGA​GCA​T-3′; GAPDH, forward: 5′-GGG​TGT​GAA​CCA​CGA​GAA​AT-3′, reverse: 5′-CCT​TCC​ACA​ATG​CCA​AAG​TT-3′; and 18S, forward: 5′-CGC​CGC​TAG​AGG​TGA​AAT​C-3′, reverse: 5′-CCA​GTC​GGC​ATC​GTT​TAT​GG-3′.

All reactions were performed in duplicate. All mRNAs of interest used the same cycling parameter: the thermal conditions consisted of an initial denaturation step at 95°C for 10 min, followed by 40 cycles of denaturation at 95°C for 15 s, annealing at 60°C for 30 s, and extension at 72°C for 30 s and a final melting curve.

### Preparation of muscle and C2C12 cell homogenates

TA muscles were collected from adult mouse hind limbs, and dissected muscles were crushed on dry ice. Muscle powder was resuspended in urea/thiourea buffer (7 M urea, 2 M thiourea, 65 mM chaps, 100 mM DTT, 10 U DNase I, and protease inhibitors [Complete; Roche/Sigma]), and protein concentration was determined using the CB-X Protein Assay kit (G-Bioscience). After trypsination, C2C12 cells were solubilized in RIPA buffer (50 mM Tris–HCl, pH 8.0, 150 mM NaCl, 1% NP-40, 0.5% sodium deoxycholate, 0.1% SDS, and protease inhibitors [Complete; Roche/Sigma]). Protein concentration was determined using the bicinchonic acid protein assay kit (Pierce/Thermo Fisher Scientific) per the manufacturer’s recommendations.

### Western blot

10–20 µg of total proteins was separated by SDS-PAGE and transferred onto nitrocellulose membranes. Nonspecific binding was blocked with 4% skim milk diluted in 1X PBS supplemented with Tween 0.1%, and membranes were incubated with primary antibodies. After thorough washing with 0.1% Tween 1X PBS, membranes were incubated with HRP-conjugated secondary antibodies (Jackson Immunoresearch Laboratories/Cederlane). After additional washes, signals were revealed using ECL substrate reagents (Thermo Fisher Scientific) and autoradiographed with x-ray films (Thermo Fisher Scientific). Quantifications were performed with the Image Lab (Bio-Rad) or ImageJ (National Institutes of Health) software.

### In vivo electroporation

Operative procedure was performed using aseptic techniques (Ravel-Chapuis et al., 2007) and according to the local ethical committee recommendations. 6-wk-old male mice were anesthetized with an intraperitoneal injection of ketamine (100 mg/kg) and xylazine (10 mg/kg) to obtain a deep state of general anesthesia. A 30-µl volume of 0.9% NaCl containing 5 µg of DNA was injected into TA muscles. Injected muscles were then electroporated with 1 cm^2^ plaque electrodes placed on each side of the leg and eight 200 V cm^−1^ pulses of 20 ms applied at 2 Hz (ECM 830; Q-Biogen).

### Behavioral tests

All injections and behavioral tests were performed in a blinded manner at the University of Ottawa Core Facility. For TubA experiments, daily injections were continued during the behavioral testing period. To minimize interference, injections were performed in the afternoon after the completion of each test, except for beam break experiments for which mice were injected before the 24-h test. Before each test, mice were habituated to the room for at least 30 min, and tests were performed under normal light conditions. Mice were handled once a day for 3 d before the first test. The beam break test is a locomotor activity test used to evaluate general motor activity and motor habituation (ambulatory activity). Each metal frame is equipped with infrared receptors and emitters. Movements of mice were video recorded for 24 h and analyzed (Omnitech Electronics). In the open-field test, mice were placed in a 45 cm × 45 cm × 45 cm box for 10 min, and their movement was video recorded and analyzed (Noldus; Ethovision). In the horizontal ladder test, mice were placed on a ladder apparatus composed of two clear Plexiglas walls (69.5 cm × 15 cm), and each wall contained 121 holes through which metal rods were spaced irregularly. Their step movement was video recorded and analyzed. The output measure of the horizontal ladder is scored through visualizing the video of the mouse walking at a slow speed and determining the number of successful steps (hit), slips/missed steps, and cheat steps. The muscle force of each animal was measured using a Grip Strength Meter (Chatillion DFE II; Columbus Instruments) with either all paws or forepaws. The mouse was moved closer to the meter until it had a firm grip on the probe. The mouse was pulled horizontally away from the bar at a speed of ∼2.5 cm/s until it released the probe. The value of the maximal peak force was recorded. This was repeated five times for each animal, with a waiting time of 10–15 s between each measurement.

Gait analysis (DigiGait) was performed using the Catwalk system (Noldus). Each mouse performed three trials during which the animal had to cross the pressure-sensitive plate of the Catwalk system without any interruption. Stride frequency, stride duration, and stance duration were recorded and averaged over the three trials. In the accelerating rotarod test, mice were placed on a rotating textured rod divided into five lanes (IITC Life Sciences). The rod rotation speed increased from 0 to 45 rpm within 1 min, with the maximal test duration set at 2 min. Each animal performed three consecutive trials with a 1-min resting interval. The latency to fall off the rod was recorded.

### Immunofluorescence microscopy and image acquisition

C2C12 cells and isolated dissociated muscle fibers were incubated with primary antibodies in PBS–0.1% Tween 20 either at room temperature for 60 min (C2C12 cells) or at 4°C overnight (isolated dissociated muscle fibers) and washed. After incubation for 1–3 h at room temperature with fluorescent secondary antibodies, NMJs were stained with α-BTX–A488 (Molecular Probes) for 2 h, and nuclei were stained with Hoechst 33258 for 10 min (Life Technologies). Coverslips were mounted on microscope slides with FluorSave reagent (Calbiochem). For accurate analysis, each image was captured on a single en-face NMJ. NMJs that were partially oblique to the field of view were only included if the oblique portion constituted less than ∼10% of the total area. Images were captured at room temperature on either a Zeiss LSM880 microscope with an AiryScan1 detector equipped with a 63× 1.4-NA or a Zeiss Axio Imager M2 Zeiss upright microscope equipped with Plan-Apochromat equipped with either a 63× 1.4-NA or a 10× 0.45 NA with AxioCam MRm charged coupled device detector. All images were processed with ZEN blue software, Zeiss AxioVision software, Photoshop CS5 (Abobe Systems), or FIJI software (ImageJ 2.0.0-rc-69/1.52n). Images were analyzed in a blinded manner by randomly renaming files with numbers using the “name_randomizer” macro in ImageJ (Osseni et al., 2016).

### Immunoprecipitation and PLA

Mouse skeletal muscle was solubilized as described above. Immunoprecipitations were then performed following the manufacturer’s instructions using the Dynabeads Protein G (Life Technologies). PLAs were performed following the manufacturer’s instructions using the Duolink anti–Mouse MINUS and anti–Rabbit PLUS In Situ PLA probes and the Duolink In Situ Detection Reagents Red (Sigma). Cells were counterstained with α-BTX–A488 for 60 min and imaged using a Zeiss LSM880 microscope with an AiryScan1 detector equipped with a 63× 1.4-NA.

### Live-cell imaging acquisition

C2C12 myotubes at 4 d old were cultured in DMEM without phenol red (Multicell) and then were incubated with α-BTX–A488 for 60 min and washed three times in DMEM without phenol red for 10 min. Images were captured on an IncuCyte ZOOM system (Sartorius). Images were captured every 30 min for 12 h at 37°C. In six-well plates, 16–25 images were taken by wells. After 24 h, α-BTX–A594 was added for 1 h. Myotubes were washed three times in DMEM for 10 min, and a final image was captured.

### Quantitative analysis of compactness and fragmentation index by “NMJ-morph”

To quantify compactness and fragmentation index, images were analyzed thanks to NMJ-morph developed in Jones et al. (2016). The compactness of AChRs at the endplate was defined as follows:

$$\mathrm{Compactness}=\left(\frac{\mathrm{AChR}\mathrm{area}}{\mathrm{endplate}\mathrm{area}}\right)\times 100.\;$$

The fragmentation index was calculated whereby a solid plaque-like endplate had an index of (0) and a highly fragmented endplate had an index that tended toward a numerical value of (1):

$$\mathrm{Fragmentationindex}=\;1-\left(\frac{1}{\mathrm{number}\mathrm{of}\mathrm{AChR}\mathrm{clusters}}\right),$$

The basic dimensions of the postsynaptic motor endplate were measured using standard ImageJ functions (Fig. S4 A). NMJ-morph was used to quantify the number of discrete AChR clusters comprising the motor endplate.

### Statistical analyses

All statistical analyses were performed using Prism 6.0 (GraphPad Software). Data are given as mean ± SEM. Student’s *t* test was used if datasets belonged to a normally distributed population with *n* > 30. Otherwise, the nonparametric two-sided *U* test (Mann-Whitney) was applied. Data distribution was assumed to be normal, but this was not formally tested. For a multiple factorial analysis of variance, two-way ANOVA was applied. P values <0.05 were considered statistically significant (shown as a single asterisk in figures, P values <5%); P values <0.01 were considered highly statistically significant (shown as two asterisks in figures, P values <1%); and P values <0.001 were considered very highly statistically significant (shown as three asterisks in figures, P values <0.1%).

### Online supplemental materials

Fig. S1 shows expression of tubulin acetylated in HEK293 cells treated with different HDAC6 inhibitors. Fig. S2 shows quantifications of numbers of AChR clusters in the presence of HDAC6 inhibitors in C2C12 cells. Fig. S3 shows the PLA in the extra-synaptic domain in TA muscle. Fig. S4 shows the flowchart realized by NMJ-morph and the effects of TubA treatment on the synaptic genes’ RNA levels and in vivo gait measurements. Fig. S5 shows the distribution of AChR area, endplate area, and diameter in mice electroporated with either mutants of HDAC6 or mutants of tubulin. Video 1 and Video 2 show AChR cluster dynamics treated with either HDAC6 inhibitors or MT drugs and imaged every 30 min for 12 h.
